# ‘GGFGGQ’ repeats in Hfq of *Acinetobacter baumannii* are essential for nutrient utilization and virulence

**DOI:** 10.1016/j.jbc.2024.107895

**Published:** 2024-10-17

**Authors:** Abhiroop Sett, Pulak Kumar Maiti, Kritika Garg, Arsalan Hussain, Snehlata Saini, Shivam Pandey, Ranjana Pathania

**Affiliations:** 1 Department of Biosciences and Bioengineering, Indian Institute of Technology, Roorkee, Uttarakhand, India; 2 Centre of Excellence in Disaster Mitigation and Management, Indian Institute of Technology, Roorkee, Uttarakhand, India

**Keywords:** post-transcriptional regulation, non-coding small RNAs, cellular energetics, pathophysiological fitness, hfq acidic tip

## Abstract

The nosocomial pathogen *Acinetobacter baumannii* is known for causing lung and soft tissue infections in immunocompromised hosts. Its ability to adapt to various environments through post-transcriptional gene regulation is key to its success. Central to this regulation is the RNA chaperone Hfq, which facilitates interactions between mRNA targets and their small RNA partners through a Sm-core domain. Notably, the *A. baumannii* Hfq protein has a uniquely long C-terminal domain with “GGFGGQ” amino acid repeats and an acidic amino acid-rich C-terminal tip (C-tip). Previous research has shown the importance of the intact C-terminal domain for Hfq's functionality. Given the significance of the C-tip in *Escherichia coli* Hfq, we examined the pathophysiological roles of the redundant ‘GGFGGQ’ repeats along with the C-tip of *A. baumannii* Hfq. We constructed several variations of Hfq protein with fewer ‘GGFGGQ’ repeats while preserving the C-tip and variants with altered C-tip amino acid composition. We then studied their RNA interaction abilities and assessed the pathophysiological fitness and virulence of genome-complemented *A. baumannii* mutants. Our findings reveal that the redundancy of the ‘GGFGGQ’ repeats is crucial for Hfq's role in pathophysiological fitness and negatively impacts *A. baumannii'*s virulence in a murine lung infection model. In addition, C-tip mutants exhibited a negative effect on both fitness and virulence, however, to a lesser extent than the other variants. These results underscore the importance of ‘GGFGGQ’ redundancy and acidic residues in Hfq's ribo-regulation and autoregulation, suggesting their critical role in establishing regulatory networks.

Over the course of last few decades, antimicrobial resistance (AMR) has turned out to be a global threat. Failure to treat bacterial infections with conventional antibiotics has led clinicians to increasingly rely on last-resort drugs. Among these infectious agents, *Acinetobacter baumannii* has been frequently found to harbor AMR genes which contribute towards their multidrug-resistant phenotype, especially against carbapenems (1, 2). Pathogenic strains of *A. baumannii* are known for causing nosocomial infections and their resilience outside the host (1, 3, 4). Previous studies on this pathogen have implicated the RNA-chaperone host factor for Qβ phage replication (Hfq) mediated post-transcriptional regulation in conferring *A. baumannii* with pathophysiological fitness (5, 6). The role of RNA chaperone Hfq is central to post-transcriptional regulation (7), facilitating interactions between small regulatory RNAs (sRNAs) and target mRNAs up to 1000 times faster than unchaperoned interactions (8).

The Hfq protein of *A. baumannii* consists of a conserved homohexameric RNA-binding Sm-domain, known as the core, and a long intrinsically disordered C-terminal domain (CTD) with unique ‘GGFGGQ’ repeats, ending with an acidic amino acid–rich C-terminal tip (C-tip) (Fig. S1, *A* and *B*) (6). The Hfq protein mediates interactions between sRNAs, ranging from 50 to 250 nucleotides, and their corresponding mRNA partners through its Sm-domain, ensuring robust regulation of pathophysiological fitness (7). In *Escherichia coli*, for instance, sRNAs facilitate approximately 2800 interactions with diverse mRNA targets, where the number of interacting mRNAs exceeds the total sRNA population (9). In *Acinetobacter* sp., various studies have demonstrated the presence of sRNAs and their targets (10, 11, 12). Post-transcriptional regulation in *Acinetobacter* sp. is known to impart regulation of genes involved in amino acid utilization, drug efflux, regulation of virulence, and biofilm formation (13, 14, 15, 16). However, our understanding of the various roles of the Hfq protein in these regulations are still in their nascent stages in *A. baumannii*.

The function of the highly conserved Hfq Sm-core is well understood owing to exhaustive research in various bacterial genera over the past few decades, especially in *E. coli* (17, 18, 19). However, only recent advances have begun to elucidate the roles of the disordered CTD of Hfq (20, 21). Functionally, certain regions of the *E. coli* Hfq CTD (66–72 amino acid residue) have been implicated in noncanonical RNA interactions (22). In *E. coli,* biophysical assays demonstrated that Hfq CTD assists in Hfq turnover by aiding in the release of the bound ds sRNA–mRNA complex (20). Thus, the CTD helps in preparing the chaperone for its next round of sRNA–mRNA interactions (20). Subsequent reports have established that the acidic amino acids on the C-tip of *E. coli* Hfq protein facilitate the release of the bound dsRNA complexes by interacting with the Sm-core, *in vitro* (21).

In a previous study, our group had shown that complete removal of the CTD along with its acidic tip in *A. baumannii* alters its RNA-binding properties and imparts a loss of pathophysiological fitness in this pathogen (6). However, given the recent insights into the functionality of Hfq CTD along with its acidic C-tip (20, 21), we wanted to garner further understanding of the *A. baumannii* CTD. The CTD of *A. baumannii* Hfq is composed of 12 tandemly repeated ‘GGFGGQ’ amino acid residues ending with a C-tip with a higher local composition of acidic amino acids compared to the *E. coli* Hfq. Hence, we sought to answer two major questions: 1. Whether the redundant ‘GGFGGQ’ residues result in functional redundancy; and 2. Is the presence of increased acidic amino acids on the C-tip is an essential component of *A. baumannii* Hfq. Towards that end, we have made several novel CTD truncations of *A. baumannii* Hfq and probed their binding affinity with RNA. Subsequently, we investigated their roles in pathophysiology and virulence by creating genome-complemented strains of *A. baumannii* harboring these mutants. Our data suggests that the ‘GGFGGQ’ repeats and C-tip are important for RNA binding and auto-regulatory aspects of *A. baumannii* Hfq. In addition, we found that these mutants exhibited compromised pathophysiological fitness, decreased growth, and reduced virulence.

## Results

### The redundant ‘GGFGGQ’ glycine repeats of Hfq CTD are conserved among the members of *Acinetobacter* sp

To investigate the degree of CTD conservation in the Hfq protein of *A. baumannii,* we performed a multiple sequence alignment of Hfq protein sequences from *A. baumannii* ATCC 17978 (WT) with different *Acinetobacter* strains and species, along with other pathogenic microbes (Fig. S2). Our result suggests that the ‘GGFGGQ’-redundancy of Hfq CTD is conserved among different strains of *A. baumannii* while exhibiting a high degree of sequence similarity with different *Acinetobacter* sp (Fig. S2). It is evident from this data that although all species of *Acinetobacter* possess a redundant GGFGGQ repeat, the overall amino acid sequence of the Hfq CTD is the same between *A. baumannii* strains compared to other species of *Acinetobacter* (Fig. S2). In addition, we observed that the CTD acidic tip of *Acinetobacter* Hfq harbors an acidic tip comprising of seven acidic amino acids compared to the four of *E. coli* Hfq protein (Fig. S2).

As *A. baumannii* is a pathogenic bacterium frequently encountered in hospital settings; hence, we considered investigating the presence of Hfq locus on the genome of clinical isolates of *A. baumannii* (23) using PCR amplification of genomic DNA from clinical strains exhibiting AMR phenotype was carried out using primers encompassing the Hfq coding sequence (Fig. S3*A*). We observed amplification bands with corresponding to the WT strain in all of our clinical strains (Fig. S3*B*). In contrast, we did not observe any amplification with these primers in other species of *Acinetobacter*. This was due to the fact that the genomic sequence of the *hfq* locus differs between *A. baumannii* and other *Acinetobacter* sp. Thus, our result indicates a probable conservation of Hfq locus along with ‘GGFGGQ’ repeats of Hfq among different clinical strains of *A. baumannii* as well.

To further investigate the level of conservation of the redundant CTD of Hfq, we cloned eight of these amplicons from the clinical strains into pUC18 plasmid and sequenced them. Our sequencing data suggests a 100% conservation of Hfq locus from the clinical strains of *A. baumannii* compared to the WT (Fig. S3*C*). Hence, the ‘GGFGGQ’ repeats along with the dense acidic tip are a characteristic feature of *A. baumannii* Hfq protein.

### Alteration in the CTD of Hfq perturbs its RNA-binding affinity

**Figure 1:**
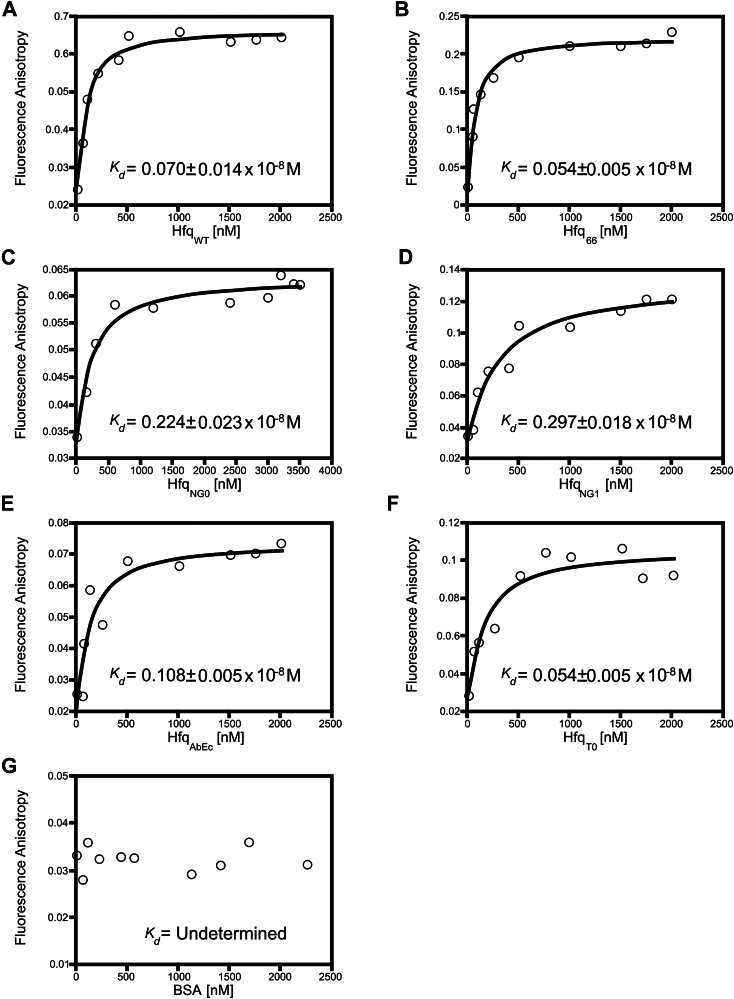
**Fluorescence anisotropy–based interaction between sRNA probe, D16-FAM, and Hfq variants.** Increasing concentration of the Hfq protein was titrated against 50 nM D16-FAM till saturation was obtained. Anisotropy reads were recorded for 200 s at 20-s intervals for each concentration of Hfq variants. The mean value of ten fluorescent anisotropy reads was plotted versus Hfq concentrations. The average *K*_*d*_ and SD of two experiments are given. Binding affinities (dissociation constant, *K*_*d*_) were determined for WT Hfq [Hfq_WT_] (*A*), Hfq_66_ (*B*), Hfq_NG0_ (*C*), Hfq_NG1_ (*D*), Hfq_AbEc,_(*E*), and Hfq_T0_ (*F*). Bovine serum albumin (BSA) was taken as a negative control (*G*), which exhibited no affinity towards the sRNA probe.

**Figure 2:**
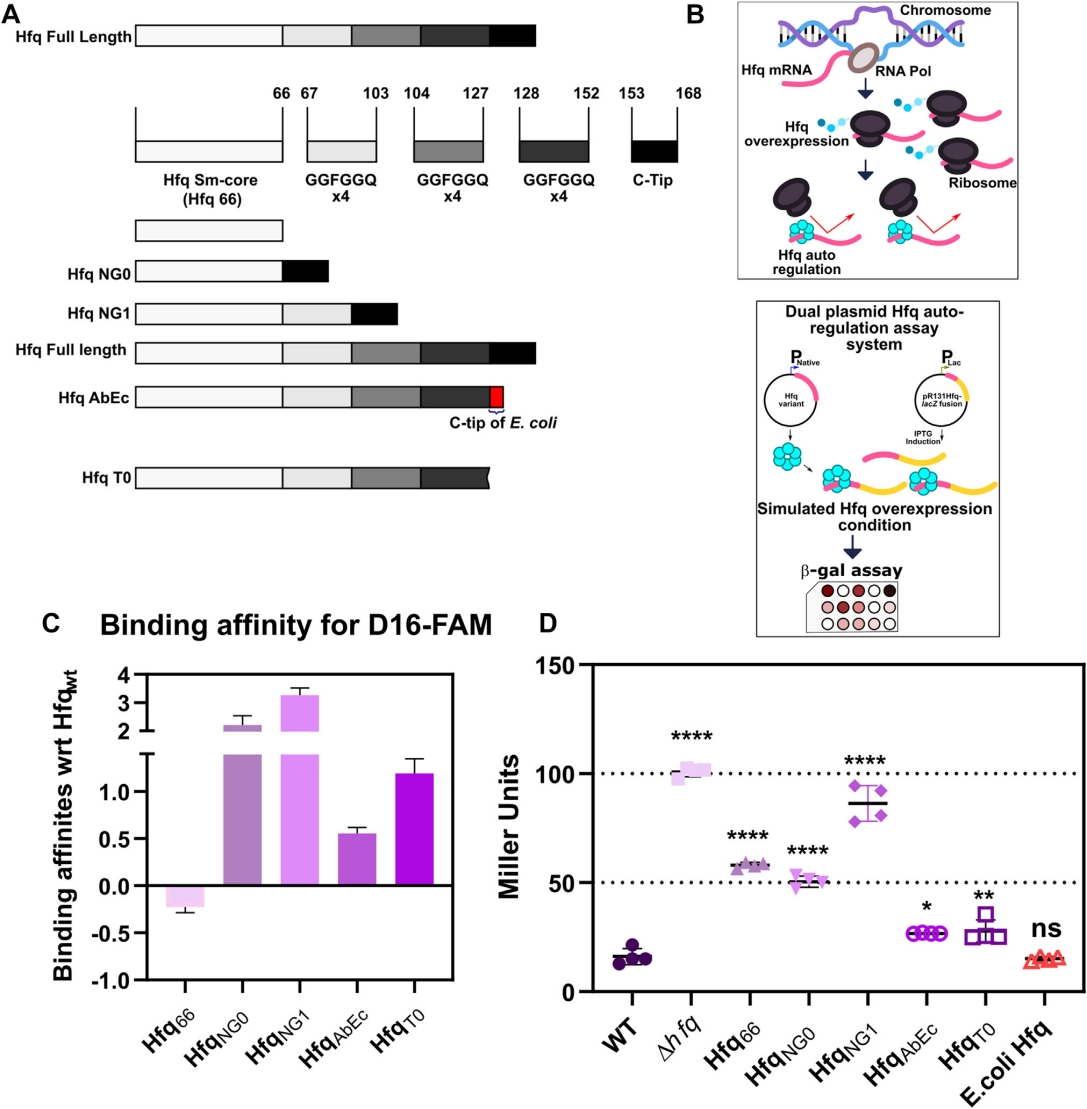
**Role of ‘GGFGGQ’ repeats and the C-tip of Hfq in auto-regulation of its own expression.***A*, schematic of the Hfq protein variants used in this study. The full length *Acinetobacter baumannii* Hfq is reimagined as different sections comprising of first 66 amino acids of the Sm-core, subsequent three regions of GGQGGF amino acid repeats, ending with the acidic C-tip. The different Hfq variants used in this study are depicted as Hfq- 66, NG0, NG1, full length, AbEc, and T0. *B*, schematic overview of the mechanism of autoregulation of Hfq. (*Upper* panel) When the Hfq protein is overexpressed within a cell, it binds to its own mRNA preventing translation. (*Lower* panel) An overview of the experimental strategy used to study Hfq auto-regulation. The ‘GGFGGQ’ and C-tip mutants are co-expressed in an *Escherichia coli* Δ*hfq* cell along with an Hfq-LacZ fusion. The expression of Hfq-LacZ fusion protein was induced by IPTG. Subsequently, the cells were incubated with chlorophenol red-β-D-galactopyranoside (CPRG) to determine the Hfq-LacZ levels *via* a β-galactosidase activity assay. *C*, relative binding affinities of the Hfq protein variants. The *K*_*d*_ values determined using fluorescent anisotropy for each of all the Hfq protein variants were normalized by that of the WT Hfq. The normalized WT value was then subtracted from the normalized values of all the mutants and plotted. *D*, the level of auto-regulation was evaluated as β-galactosidase activity in terms of Miller units and compared. Each data represents the mean of four independent experiments, and the error bars represent S.D. Statistical significance was determined by one-way ANOVA (*p*-value was ∗, *p* ≤ 0.05; ∗∗, *p* ≤ 0.001; ∗∗∗∗, *p* ≤ 0.0001; ns, non-significant). Tukey’s test was used as a *post hoc* test to determine the statistical significance of all pairs of data.

Given this high degree of conservation of Hfq CTD, we wanted to investigate the role of the redundant ‘GGFGGQ’ repeats and the acidic tip in RNA binding (Fig. 1). We constructed Hfq CTD truncated variants on pET28 (Fig. 2*A*) vector and expressed and purified them from a heterologous *E. coli* host (Fig. S4). One of the mutant proteins that we used was from our previous study; Hfq_66_ (66 amino acids) which contained only the core Sm domain (6). The other mutants constructed for this study included; Hfq_NG0_ (66 amino acids + acidic tip or C-tip) consisting of the Hfq core along with the last 18 amino acids of the C-tip; Hfq_NG1_ (103 amino acids + C-tip) consisting of Hfq core with four ‘GGFGGQ’ repeats along with the C-tip; Hfq_AbEc_ (152 amino acid + *E.coli* hfq C-tip) consisting of all the ‘GGFGGQ’ repeats with the native 18 amino acids of the C-tip replaced with six C-tip amino acids from *E. coli* Hfq; and HfqT0 (152 amino acids and no C-tip) consisting of all the ‘GGFGGQ’ repeats without any acidic tip.

It has been observed that the complete removal of Hfq CTD results in changes in binding affinities towards RNA (6). Since our Hfq variants do possess some parts of the CTD, we wanted to explore their RNA-binding affinities as well. We used a fluorescently labeled sRNA probe, D16-FAM, which was previously reported to interact with *E. coli* Hfq (21). We titrated increasing concentration of Hfq protein variants until saturation was reached and monitored RNA-binding by measuring increase in fluorescence anisotropy in order to determine the binding affinities (Fig. 1). We found that the complete removal of the ‘GGFGGQ’ repeats and the C-tip (Hfq_66_) results in an increase in RNA-binding affinity (reduction in *K*_*d*_ -value) of the protein. As the Sm-core is devoid of any CTD, it may contribute towards tighter binding with RNA molecules. Whereas, reduction in proximity of the C-tip to the core reduces the binding affinity (increase in *K*_*d*_ -value) in the case of Hfq_NG0_ and Hfq_NG1_ (Fig. 2*C*). This could be attributed to the fact that the C-tip is essential in Hfq turnover, thus, its presence and proximity to the Sm-core may dictate the binding affinities in these variants (20, 21). In case of the C-tip mutants (Hfq_T0_ and Hfq_AbEc_), alteration of the acidic C-tip causes these variants to exhibit reduced affinity towards the sRNA probe; however, the *K*_*d*_ values were lower than the Hfq_NG0_ and Hfq_NG1_. Hence, alterations in the CTD length and C-tip composition introduce perturbations in RNA-binding affinities of different Hfq variants.

### The redundant glycine repeats are involved in autoregulation of Hfq expression

It has been established previously that the CTD of *E. coli* Hfq is responsible for the auto-regulatory role of Hfq (24). The intact CTD of *E. coli* and *A. baumannii* Hfq was found to be crucial in negative autoregulation of its own translation by binding to its mRNA (6) (Fig. 2*B*). Results from our previous experiments suggest that Hfq mutants exhibit altered affinity towards RNA molecules. So, we wanted to check whether the redundancy in ‘GGFGGQ’ repeats or alteration of acidic amino acid residues on the C-tip had any effect on the auto-regulatory aspects of *A. baumannii* Hfq. To investigate the same, we co-expressed the Hfq variants and a fusion protein of Hfq-*lacZ* in an *E. coli Δhfq* strain. In order to simulate Hfq overexpression conditions, the Hfq-*lacZ* expression was induced with IPTG (Fig. 2*B*). These cells were then incubated for 8 h to allow for repression of β-galactosidase activity resulting from translation repression of Hfq-*lacZ* synthesis due to interaction between the Hfq variants and Hfq-*lacZ* mRNA. We observed that, in comparison to the WT, alterations in the ‘GGFGGQ’ repeats (Hfq_66_, Hfq_NG0_, Hfq_NG1_) resulted in a significant loss of *lacZ* repression whereas the change was less significant in the case of C-tip mutants (Hfq_AbEC_ and Hfq_T0_) (Fig. 2*D*). This observation indicates that although the density and composition of acidic C-tip are important for Hfq to interact with its own mRNA, the conserved length of the CTD, conferred by the ‘GGFGGQ’ repeats, is much more essential for the same. Hence, the polymeric nature of the intrinsically disordered Hfq CTD conferred by the glycine repeats is a critical component of Hfq auto-regulation.

### The redundancy of Hfq CTD plays a critical role in regulating growth in varied carbon sources

The observations from our previous experiments implicate the C-terminal glycine repeats and the acidic C-tip residues of Hfq in its ability to exhibit optimal RNA-binding affinity and auto-regulating its mRNA. These aspects of the Hfq protein help in establishing a post-transcriptional regulatory network. Hence, we wanted to examine how these variants affect the pathophysiological fitness of *A. baumannii in vivo*. We integrated the genetic sequence of *hfq* variants cloned in pET28 overexpression vector into the native genomic locus of *hfq* in the *A. baumannii* Δ*hfq* strain using a previously described homologous recombination-based approach (23, 25) (Fig. S5).

**Figure 3:**
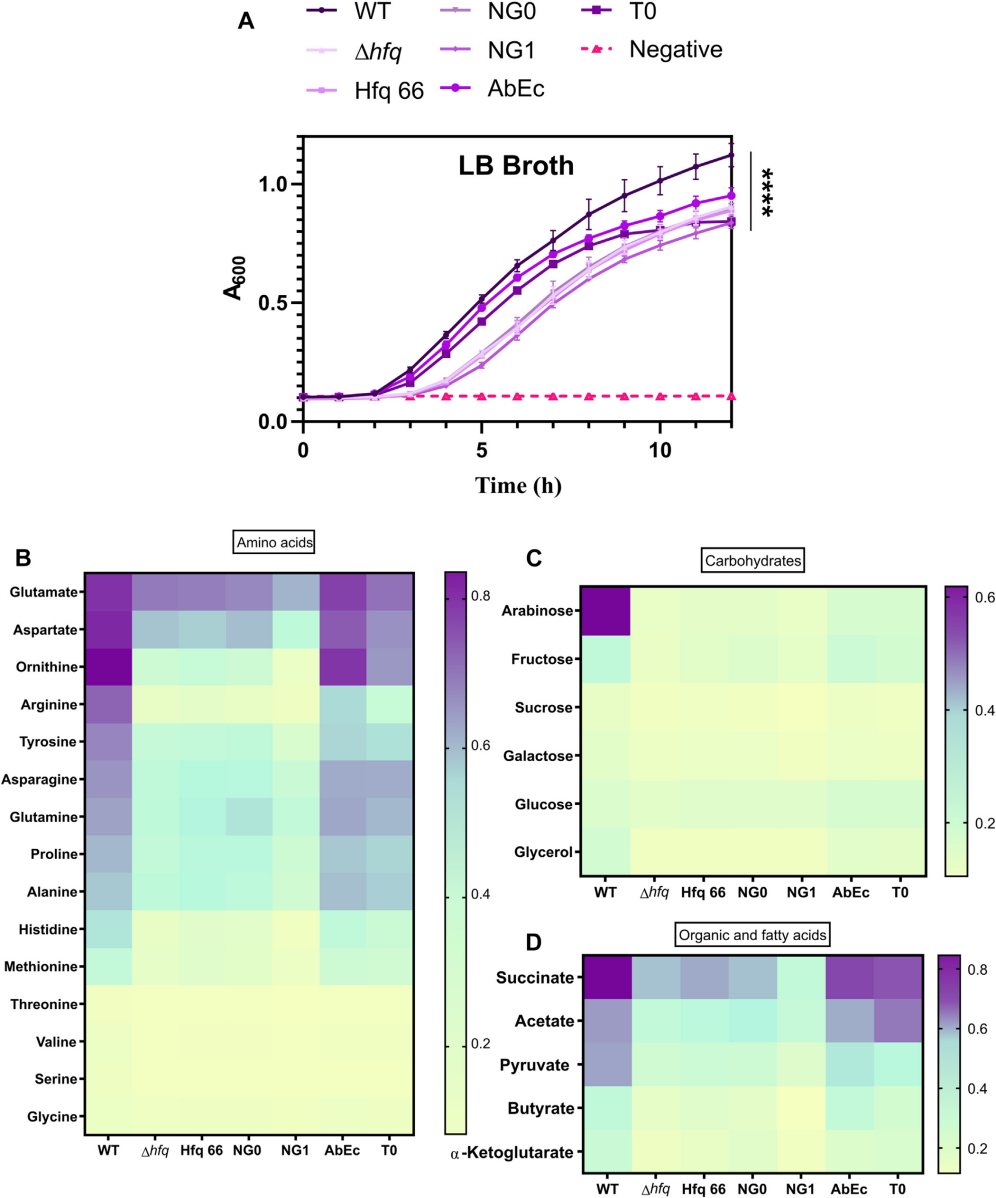
**The effect of Hfq deletion and subsequent genomic complementation by constructs carrying varying CTD, on growth of *A. baumannii*.***A*, the growth profile of all the *A. baumannii* strains. The *A. baumannii* strains were grown in a 96-well plate at 37 °C with shaking, and the growth was monitored by measuring the optical density at 600 nm in a multimode plate reader at one-hour intervals. Each point represents the mean of four values with SD shown as error bars. Statistical significance was determined by one-way ANOVA, and *p* value was ∗∗∗∗, *p* ≤ 0.0001. Tukey’s test was used as a *post hoc* test to determine statistical significance between all pairs of end point data. *B*, the end-point of growth in different amino acids is represented as a heat map. The variants of *A. baumannii* were grown in M9 minimal salts supplemented with 10 mM of the indicated amino acids (shown on the *left*) as a sole carbon source. The end-point optical density at 600 nm after 12 h of growth of each variant has been represented as varying colors ranging from a darker shade of *blue* (increased growth) to a lighter shade of *blue* (low growth). Different amino acids are indicated on the *left* and the different *A. baumannii* strains are depicted at the *bottom* from *left* to *right*. *C*, the end-point of growth in different carbohydrates is represented as a heat map. The variants of *A. baumannii* were grown in M9 minimal salts supplemented with 10 mM of the indicated carbohydrates (shown on the *left*) as the sole carbon source. The end-point optical density at 600 nm after 12 h of growth of each variant has been represented as varying colors ranging from a darker shade of *blue* (increased growth) to a lighter shade of *blue* (low growth). Different carbohydrates are indicated on the *left* and the different *A. baumannii* strains are depicted at the *bottom* from *left* to *right*. *D*, the end-point of growth in different organic and fatty are represented as a heat map. The variants of *A. baumannii* were grown in M9 minimal salts supplemented with 10 mM of the indicated organic and fatty acids (shown on the *left*) as a sole carbon source. The end-point optical density at 600 nm after 12 h of growth of each variant has been represented as varying colors ranging from a darker shade of *blue* (increased growth) to a lighter shade of *blue* (low growth). Different organic and fatty acids are indicated on the *left* and the different *A. baumannii* strains are depicted at the *bottom* from *left* to *right*. For *B*, *C*, and *D*, each value on the heat map represents the mean of optical density at 600 nm of four values at the end of 12 h of growth. All the growth phenotype assays are the representative plot of three independent biological replicates.

The loss of Hfq has been shown to induce a slow growing phenotype in different bacterial strains (6, 26, 27). Hence, we profiled the growth phenotype of these genome-complemented mutant strains of *A. baumannii,* harboring *hfq* variants, in Lysogeny broth (LB broth). The deletion mutant of Hfq, Hfq core, and the mutants with shortened glycine repeats (*Δhfq*, Hfq_66_, Hfq_NG0_, Hfq_NG1_) displayed pronounced growth defect in LB when compared with the WT (Fig. 3*A*). However, the mutations on the C-tip (Hfq_AbEC_ and Hfq_T0_) resulted in intermediate growth perturbations compared to WT and the rest of the mutants. This emphasizes the fact that changes in the local acidic amino acid residue composition and glycine repeats alter the optimal growth potential of *A. baumannii.*

As it was previously determined that CTD as a whole is necessary for optimal utilization of different carbon sources (6). We wanted to further examine the growth kinetics of the CTD mutants in minimal salts supplemented with different amino acids, carbohydrates, and organic acids. According to different multiomics studies, amino acid utilization genes are more abundant in *A. baumannii* than carbohydrate utilization genes (28, 29). In addition, the levels of different amino acids like glutamate and arginine are reported to be upregulated in a murine lung infection model of *A. baumannii.* (30). As evident from the growth kinetics (Fig. S6), the CTD mutants exhibit growth fitness reminiscent of that observed in LB broth when grown in the presence of amino acids (Fig. 3*B*). Hence, Hfq with its unusual CTD may aid this pathogen to utilize bioavailable amino acids during the course of an infection.

Another key feature of *A. baumannii* is the absence of the glycolytic enzyme hexokinase and a phosphosugar transfer system for glucose (28). In addition, carbohydrate uptake and utilization pathways are less abundant in this pathogen compared to that of amino acids (28, 29). Hence, these factors render the pathogen incapable of optimally utilizing different simple sugars, especially *via* the glycolytic pathway. So, we further evaluated how the perturbations in Hfq impact the ability of *A. baumannii* to utilize different carbohydrates. Our results show that, *A. baumannii* WT and mutants are unable to optimally utilize different carbohydrate sources (Fig. 3*C*). However, the WT cells were able to utilize arabinose which was not the case with any of the other mutants (Fig. 3*C*). It was recently observed that the pathogenic *A. baumannii* can utilize arabinose using a previously uncharacterized operon system that differs from that of *E. coli* and is absent in *Acinetobacter baylyi* (31). An important feature of this novel arabinose utilization pathway is that it converts arabinose to α-ketoglutarate, an intermediate of the TCA cycle, hence bypassing glycolysis (31). Our results highlight that, any perturbations of the *Acinetobacter baumanii* Hfq CTD render the pathogen unable to utilize arabinose. Hence, this indicates that the Hfq protein, with its redundant glycine-rich CTD and C-tip, either directly or indirectly regulates this pathway. Further investigation into the same may reveal novel regulatory networks behind arabinose metabolism.

An important observation from our results is that perturbations in the Hfq CTD result in growth defects in amino acids (Fig. 3*B*). Catabolism of amino acids involves the formation of keto acids and other TCA cycle intermediate. Hence, we further profiled the growth of our *A. baumannii* strains in organic acids like succinate, pyruvate, α-ketoglutarate, and acetate, which are catabolized *via* the TCA cycle. In all of these sources, we found that disruption of the CTD imparted a growth defect, which was much more pronounced in the case of ‘GGFGGQ’ mutants than that of the C-tip mutants (Fig. 3*D*). With α-ketoglutarate, an intermediate product of arabinose catabolism in *A. baumannii,* we did not observe any similarity with the growth pattern in arabinose (Fig. S6). Instead, growth pattern in α-ketoglutarate resembled that of the other TCA cycle intermediates (Fig. S6). The TCA-cycle intermediate acetate is also classified as a short chain fatty acid (SCFA) that gets converted into acetyl CoA before entering the TCA cycle (32, 33, 34). So, we also included butyrate as another representative SCFA in our assay to see whether alterations in the CTD result in changes in growth in these carbon sources. Our results show that these SCFAs, along with other organic acids, can support the growth of WT cells, however, not to the extent observed with succinate (Fig. 3*D*). The C-tip mutants were able to survive better in these sources compared to the other mutants (Fig. 3*D*), especially in acetate, where they exhibited a better growth phenotype than the WT (Fig. S6). Hence, loss of redundancy in glycine repeats of Hfq renders the pathogen inefficient in its ability to utilize specific carbon sources with probable ramifications in its overall ability to infect the host.

### GGFGGQ repeats are indispensable as they play a critical role in cellular stress tolerance

**Figure 4:**
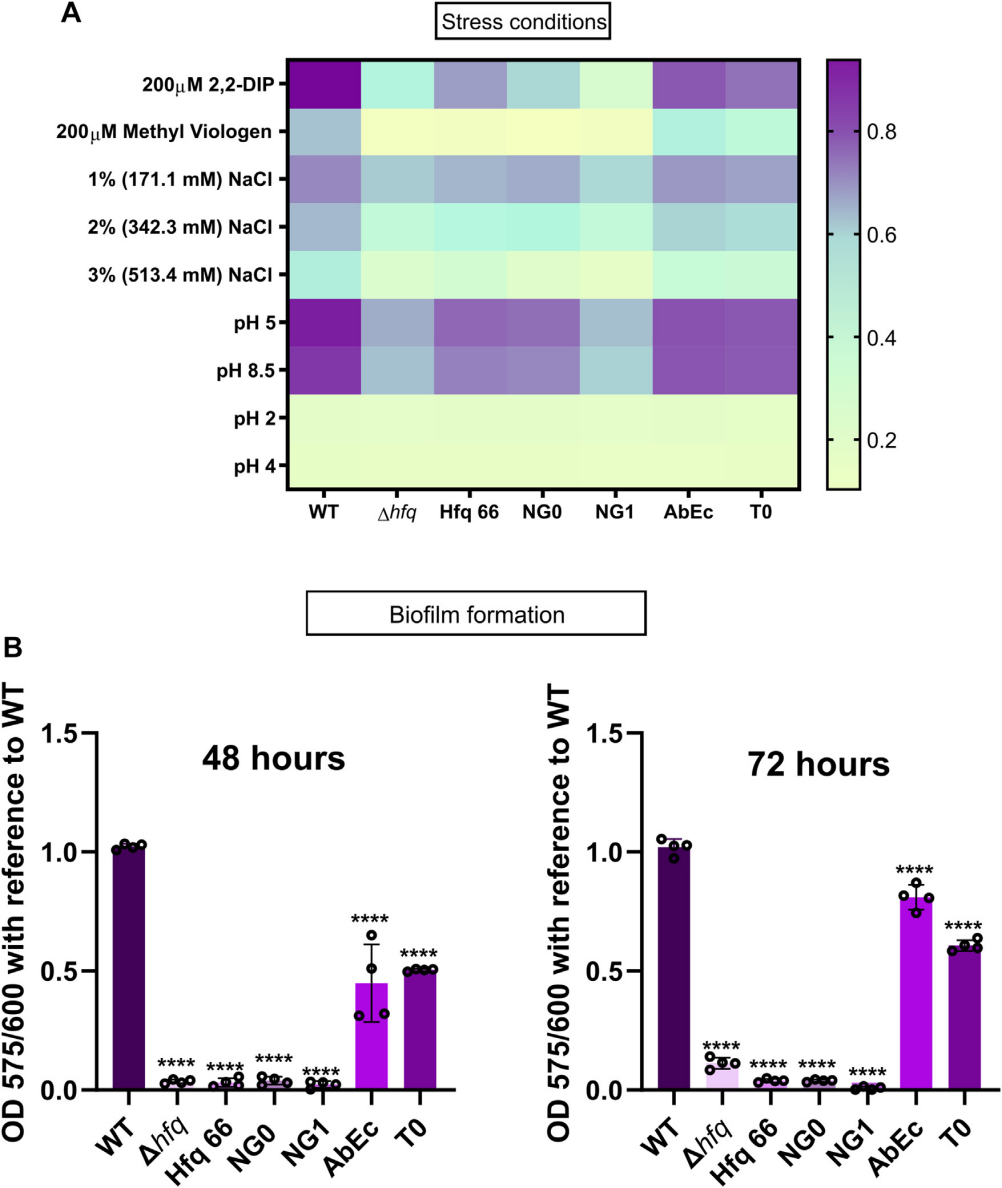
**Impact of alteration of the ‘GGFGGQ’ repeats and C-tip composition of Hfq on the ability of *A. baumannii* to tolerate stress and form biofilms.***A*, the end-point growth heat map of all the variants in different stress conditions. All the variants of *A. baumannii* were grown in LB broth in presence of the indicated stress conditions; iron limitation (LB broth supplemented with 200 μM 2,2-dipyridyl [DIP]), oxidative stress (LB broth supplemented with 200 μM methyl viologen), osmotic stress (LB broth supplemented with additional 1% (171.1 mM), 2% (342.3 mM), and 3% (513.4 mM) NaCl), and pH stress (pH of the LB media adjusted to pH 2, pH 4, pH 5, and pH 8.5 with HCl). The end-point optical density at 600 nm after 12 h of growth of each variant has been represented as varying colors ranging from a darker shade of *blue* (increased growth) to a lighter shade of *blue* (low growth). Each value on the heat map represents a mean optical density of four values at the end of 12 h of growth. All the growth phenotype assays are the representative plot of three independent biological replicates. *B*, biofilm formation assay. Actively growing cells of the WT and Hfq variants of *A. baumannii* were inoculated into LB broth and aliquoted into 12-well plates. These cultures were incubated at 30 °C in static conditions for 48 and 72 h, and the optical density was read at 600 nm. The wells were thoroughly washed, stained with fresh crystal violet, and washed again. The stained biomass was dissolved in 30% acetic acid and quantified by measuring optical density at 575 nm. Relative biofilm formation was determined by calculating the ratio of A575 and A600 and then plotted w.r.t WT. Each bar represents the mean of four experiments, and the error bars represent S.D. Statistical significance was determined by one-way ANOVA, and the *p*-value was ∗∗∗∗, *p* ≤ 0.0001. Tukey’s test was used as a *post hoc* test to determine the statistical significance of all pairs of data.

During the course of infection, a pathogenic bacterium experiences a plethora of host-induced stress conditions. For example, it is exposed to acidic environments of a phagosome, reactive oxygen species from host immune cells and metal ion limitation induced by host nutritional immunity (35, 36, 37). During an invasion by a pathogenic bacterium, the host reprogrammes its iron homeostasis modules to sequester free iron (36). This reprogramming results in lowered intracellular iron levels in macrophages and expression of iron chelating factors like calprotectin, neutrophil gelatinase-associated proteins lactoferrin, and intracellular iron storage protein ferritin (36). To garner insight into the impact of pH alterations, oxidative stress, and iron limiting conditions, we subjected them to grow in varied pH ranges, oxidative stress *via* methyl viologen, and in presence of an iron chelator 2,2′-dipyridyl, respectively. The redundancy in the ‘GGFGGQ’ amino acid was found to be a major contributor towards imparting growth fitness under these physiologically relevant stress conditions, We also observed that all the strains including the WT were unable to exhibit optimal growth at an extremely acidic pH 4 and pH 2 (Fig. 4*A*). Finally, we experimentally determined whether salinity-induced osmotic pressure is mitigated by the pleiotropic physiological regulatory networks established by the Hfq protein by assessing the mutant strains for end-point growth in high concentrations of NaCl. In line with our previous observations, the removal or perturbations in the glycine repeats result in a growth defect in osmotic stress while changes in the composition of the acidic tip did not translate to such pronounced growth defect compared to the other mutants and the WT (Fig. 4 *A*).

In view of these observations, we wanted to explore how the glycine repeats affect Hfq-mediated fitness. Consequently, we assessed the biofilm forming ability of the WT and the mutants, as this phenomenon aids bacterial cell populations in evading adverse conditions both within and outside its host (38, 39). We subjected these strains to biofilm-forming static growth conditions over a period of 48 h and 72 h. We found a similar trend in the biofilm-forming potential of these mutants as was exhibited in nutrient utilization and stress tolerance assays (Fig. 4*B*). In a previous report by Kuo *et al.*, it was determined that the deletion of *A. baumannii* Hfq lowers the CsuA/B pili gene transcript (5). As the Csu operon and the BfmRS two component system has been linked to biofilm formation in *A. baumannii* (1, 40, 41), we evaluated the transcript levels of *csuA/B*, *csuD* and *csuE, bfmR and bfmS* by qPCR in mid-log phase cells of *A. baumannii* WT and mutants. The *csu* pili genes were significantly downregulated in the mutants, with the highest downregulation observed in the case of Hfq core and mutants with shortened glycine repeats (Hfq_66_, Hfq_NG0_, Hfq_NG1_) (Fig. S7*A*). A similar trend was observed in the case of *bfmR and bfmS* genes (Fig. S7*A*). Since our data is in agreement with a previous report by Kuo *et al.* (5), it emphasizes the fact that loss of *hfq* cannot be complemented with Hfq proteins that possess an altered CTD even with their C-tip intact. Hence, our results implicate the need for a full-length *A. baumannii* Hfq in stress mitigation and biofilm formation by this pathogen.

### Disruption of cellular energetics in the CTD mutants of Hfq contributes to loss of fitness

**Figure 5:**
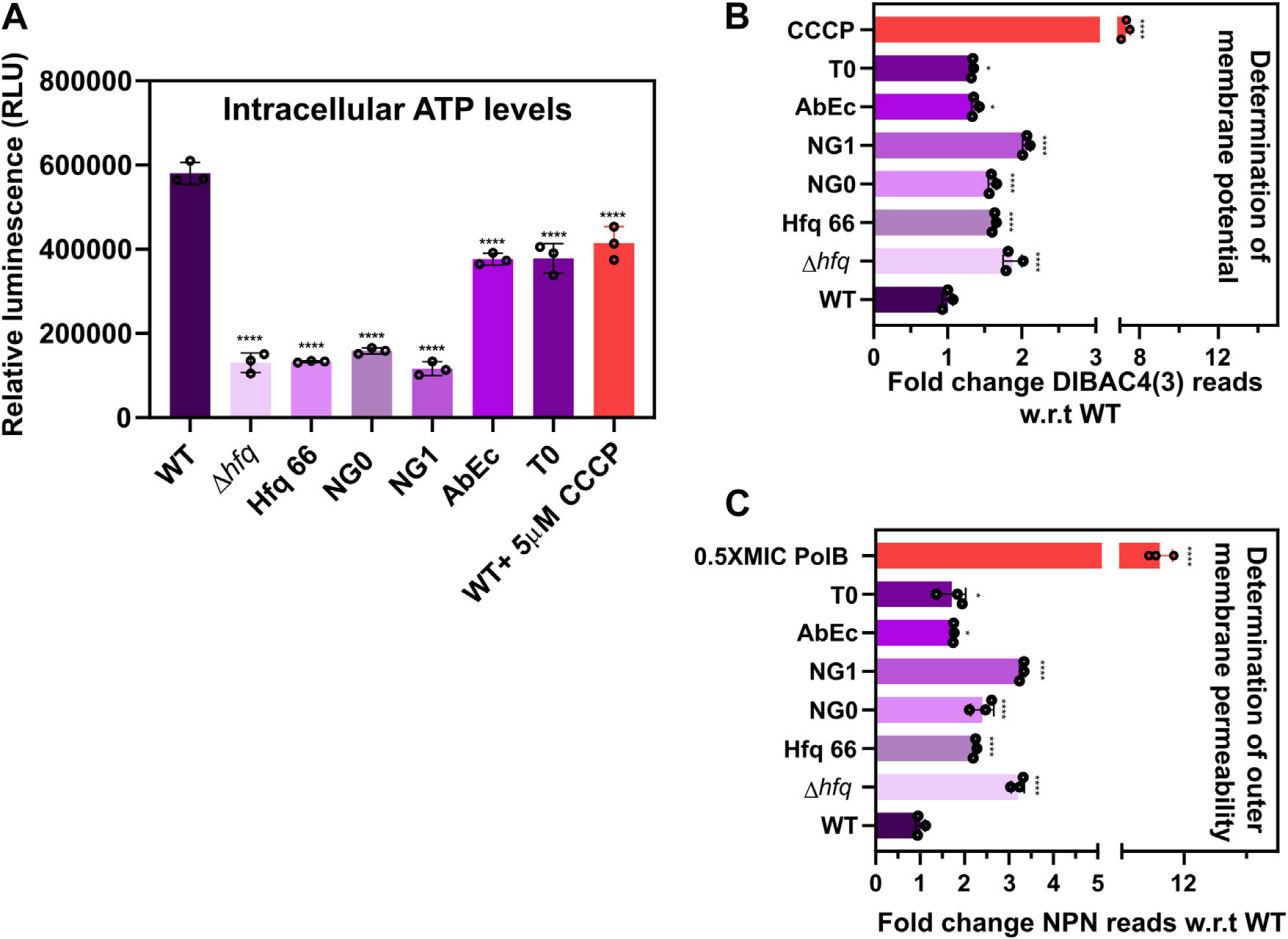
**Modifications in glycine repeats and the C-tip affects membrane stability and intracellular ATP content of *A. baumannii.****A*, determining the intracellular ATP levels. Reduction in intracellular ATP levels in the *A. baumannii* WT and Hfq variants grown in LB broth using a luciferase/luciferin assay kit with 5 μM CCCP-treated WT cells as positive control. The relative fold change in the luminescence (RLU) was calculated by dividing the luminescence readout with the optical density of the cells at 600 nm. Each bar represents the mean of three experiments, and the error bars represent the SD. Statistical significance was determined by one-way ANOVA, and the *p*-value was ∗∗∗∗, *p* ≤ 0.0001. Tukey’s test was used as a *post hoc* test to determine the statistical significance of all pairs of data. *B*, quantifying the degree of membrane depolarization. Measurement of the membrane potential component Δψ was performed using the membrane potential–sensitive fluorescent probe DiBAC4. Actively growing variants of *A. baumannii* and WT cells were subjected to the DiBAC4 fluorescent dye–based assay and the fluorescence was normalized against optical density at 600 nm. The normalized values were plotted w.r.t the WT cells. WT cells treated with 5 μM CCCP were taken as a positive control. Each bar represents the mean of three experiments, and the error bars represent the SD. Statistical significance was determined by one-way ANOVA (*p*-value was ∗, *p* ≤ 0.05; ∗∗∗∗, *p* ≤ 0.0001). Tukey’s test was used as a *post hoc* test to determine the statistical significance of all pairs of data. *C*, evaluating the degree of outer membrane disruption. Actively growing *A. baumannii* and Hfq mutant cells were probed with N-phenyl-1-napthylamine (NPN) to assess the outer membrane permeability of these strains. The fluorescence reading of the NPN dye upon encountering a permeable outer membrane was normalized against optical density at 600 nm. The normalized values were then plotted w.r.t WT cells. We treated WT cells with 0.5X MIC (0.125 mg/L) of polymyxin B as a positive control. Each bar represents the mean of three experiments, and the error bars represent the SD. Statistical significance was determined by one-way ANOVA (*p*-value was ∗, *p* ≤ 0.05; ∗∗∗∗, *p* ≤ 0.0001). Tukey’s test was used as a *post hoc* test to determine the statistical significance of all pairs of data.

In all of our growth profiling assays, we observed a similar pattern; the Hfq mutants with alteration in their ‘GGFGGQ’ repeats exhibited the highest growth defect followed by the C-tip mutants. Hence, we wanted to delve into the causal factors contributing to this observation. A cell, during its active growth phase, requires optimal energy in the form of ATP. So, we determined the intracellular ATP levels of WT and mutant cells of *A. baumannii* grown in LB broth using a luciferase/luciferin assay. We used WT cells treated with CCCP as a positive control as it is known to deplete ATP levels within a bacterial cell (23). We found that the *A. baumannii* cells with Hfq_66_ and reduced ‘GGFGGQ’ repeats (Hfq_NG0_, Hfq_NG1_) exhibited the highest significant fold reduction in intracellular ATP levels comparable to the Hfq deletion mutant strain (Fig. 5*A*). Changing the local composition of the acidic amino acid residues (Hfq_AbEc_, Hfq_T0_) also significantly lowered ATP levels (Fig. 5*A*). However, ATP levels in these two mutants were comparable to WT cells treated with 5 μM CCCP (a proton motive force (PMF) disruptor) and not to the extent observed in the case of the other mutants (Fig. 5*A*). Hence, the observed growth disruptions in the mutants could be attributed to their intracellular reduction in ATP levels.

As the mutants exhibited lowered intracellular ATP, we wanted to evaluate other factors which may contribute towards this observation. A key component of the bacterial cell that takes center stage in ATP production is the PMF. Hence, we assessed if there is any disruption in the PMF of the cell using an anionic lipophilic dye, DiBAC4. This dye enters a bacterial cell with a depolarized membrane resulting in increased fluorescence, which is indicative of an altered Δψ component of the PMF. As a positive control, we treated the WT cells with CCCP, as it disrupts the PMF gradient to lower intracellular ATP levels. Our results are indicative of enhanced depolarized membranes compared to the WT, especially in the absence of the conserved glycine repeats (Fig. 5*B*). Membrane polarization can be affected due to alterations in the membrane integrity (42, 43). Moreover, the Hfq protein of *E. coli, Salmonella* sp, and *A. baumannii* are known to be involved in maintaining membrane homeostasis (5, 44, 45). This led us to investigate the outer membrane integrity of all the *A. baumannii* strains using N-phenyl-1-napthylamine dye that exhibits increased fluorescence upon encountering a permeable outer membrane. We used a sub-MIC concentration of polymyxin B–treated WT cells as a positive control. We observed that a similar trend is followed by all the mutants as was observed in the DiBAC4 assay (Fig. 5*C*). It can be so reasoned that the loss of membrane homeostasis leads to disruption of PMF contributing towards the lowered ATP.

In *E. coli* and *Salmonella* sp., loss of membrane integrity is sensed by a multicomponent pathway where Hfq plays a key role (44, 45) (Fig. S7*C*). In *E. coli,* loss of outer membrane integrity is marked by the upregulation of misfolded outer membrane proteins (OMPs) which triggers the activation of σ^E^ (RpoE) (45). The σ^E^ initiates the transcription of chaperone and repair proteins along with different sRNAs (45). These sRNAs, along with Hfq, lower the OMP levels and restore membrane integrity, eventually lowering the active σ^E^ levels (Fig. S7*C*) (45). Hence, the loss of Hfq constitutively turns on the σ^E^-mediated pathway, resulting in an upregulation in OMP levels (44, 46, 47, 48). It was previously found out that deletion of Hfq results in the upregulation transcripts of the OMP CarO in *A. baumannii* (5). Therefore, we used qPCR to determine the transcript levels of *rpoE* and OMPs like *omp33*, *carO*, *dcaP*, and *oprD* genes of *A. baumannii* (49, 50, 51). We found that the transcript levels of *rpoE* and OMP genes are upregulated in the mutants (Fig. S7*B*). These observations are in agreement with the known pathways of membrane homeostasis involving Hfq in other bacterial genera (44, 45). As the genetic markers for membrane homeostasis were perturbed, we wanted to evaluate whether it also leads to any morphological changes in *A. baumannii* cells. Therefore, we imaged mid-log phase *A. baumannii* WT and mutant cells using scanning electron microscopy. We observed that all the Hfq mutants with reduced ‘GGFGGQ’ repeats and the Hfq core are morphologically altered exhibiting enhanced pit formation compared to the WT and C-tip mutants (Fig. S8). Hence, our results indicate that the conservation of the ‘GGFGGQ’ repeats is essential for maintaining membrane homeostasis, PMF, and intracellular ATP levels of *A. baumannii,* overall contributing towards conferring physiological fitness.

### Reducing the number of the ‘GGFGGQ’ repeats of Hfq CTD leads to decreased virulence

**Figure 6:**
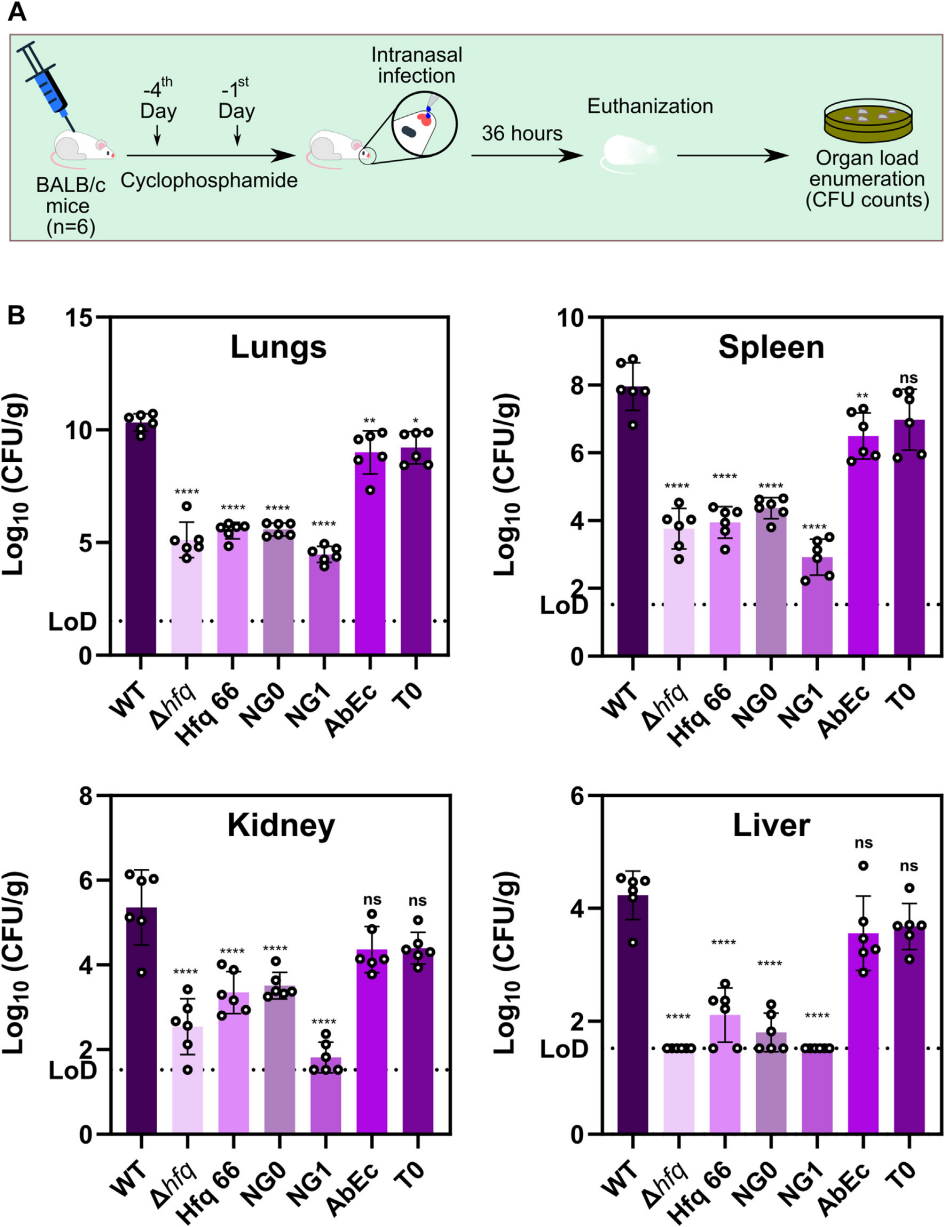
**Assessment of pathogenesis in a murine lung infection model.***A*, general scheme of establishing a murine lung infection model by *A. baumannii* WT and Hfq mutant strains. The quarantined mice (n = 6) were injected with cyclophosphamide on −4 days and −1 day before infection. Subsequently, the mice were infected with *A. baumannii* WT and variants *via* intranasal route. Post 36 h of infection, the mice were euthanized and bacterial organ burden was enumerated. *B*, determination of organ load of bacterial cells. The indicated organs isolated from the infected mice were homogenized in sterile 1X PBS. The organ burden was enumerated by counting CFUs after plating serial dilutions of the organ homogenate on agar plates. The error bars represent SD. Statistical significance was determined by one-way ANOVA (*p*-value was ∗, *p* ≤ 0.05; ∗∗, *p* ≤ 0.001; ∗∗∗∗, *p* ≤ 0.0001; ns, non-significant). Tukey’s test was used as a *post hoc* test to determine the statistical significance of all pairs of data.

The pathogenic *A. baumannii* is known to cause various ailments in the human host including pneumonia in critically ill patients (1). We have demonstrated that the alterations in the ‘GGFGGQ’ repeats or local acidic amino acid composition of the C-tip of Hfq renders the pathogenic *A. baumannii* sensitive to iron limitation, pH alterations, and osmotic stress (Fig. 4*A*). In addition, these mutants exhibit a characteristic loss of growth fitness and lowered biofilm compared to the WT (Figs. 3 and 4*B*). As per previous reports, the deletion of *hfq* rendered this pathogen less virulent in a systemic murine infection model (6). Therefore, we wanted to investigate the ability of *A. baumannii* Hfq mutants to establish a lung infection and survive within its host. Towards the same, we established a murine pneumonia infection model by infecting BALB/c mice (n = 6) with the WT and mutant strains (Fig. 6*A*). We observed that all the strains were able to establish lung infection (Fig. S9), however, the mutants exhibited a lowered lung CFU count than the WT (Fig. 6*B*). To check how well the WT and the mutant strains migrate to other organs during infection, we enumerated CFU counts in spleen, kidney, and liver. Overall, we found that the Sm-core (Hfq_66_) and perturbations in the ‘GGFGGQ’ redundancy (Hfq_NG0_, Hfq_NG1_) reduced the organ burden of the pathogen. As a result, these *hfq* mutations make *A. baumannii* less virulent, in both lungs and other organs (Fig. 6*B*). However, alteration in the local C-tip acidic amino acid composition (Hfq_AbEc_, Hfq_T0_) also reduced the virulence but not to the extent observed in the case of the other mutant strains (Fig. 6*B*). The CFU counts of the C-tip mutants were lower than the WT but more than the rest of the mutants especially in the lungs. We found that although the mean CFU count was lower than the WT for the C-tip mutants in spleen, kidney, and liver, the difference was found to be statistically nonsignificant (Fig. 6*B*). This could be due to the fact that the alterations in C-tip do not result in an extensive loss of fitness compared to the WT, as was seen in our previous results. Hence, Hfq, with its redundant CTD and native C-tip, is essential for *A. baumannii* to optimally maintain multiple facets of pathophysiology, culminating in its ability to cause infection in its host.

## Discussion:

**Figure 7:**
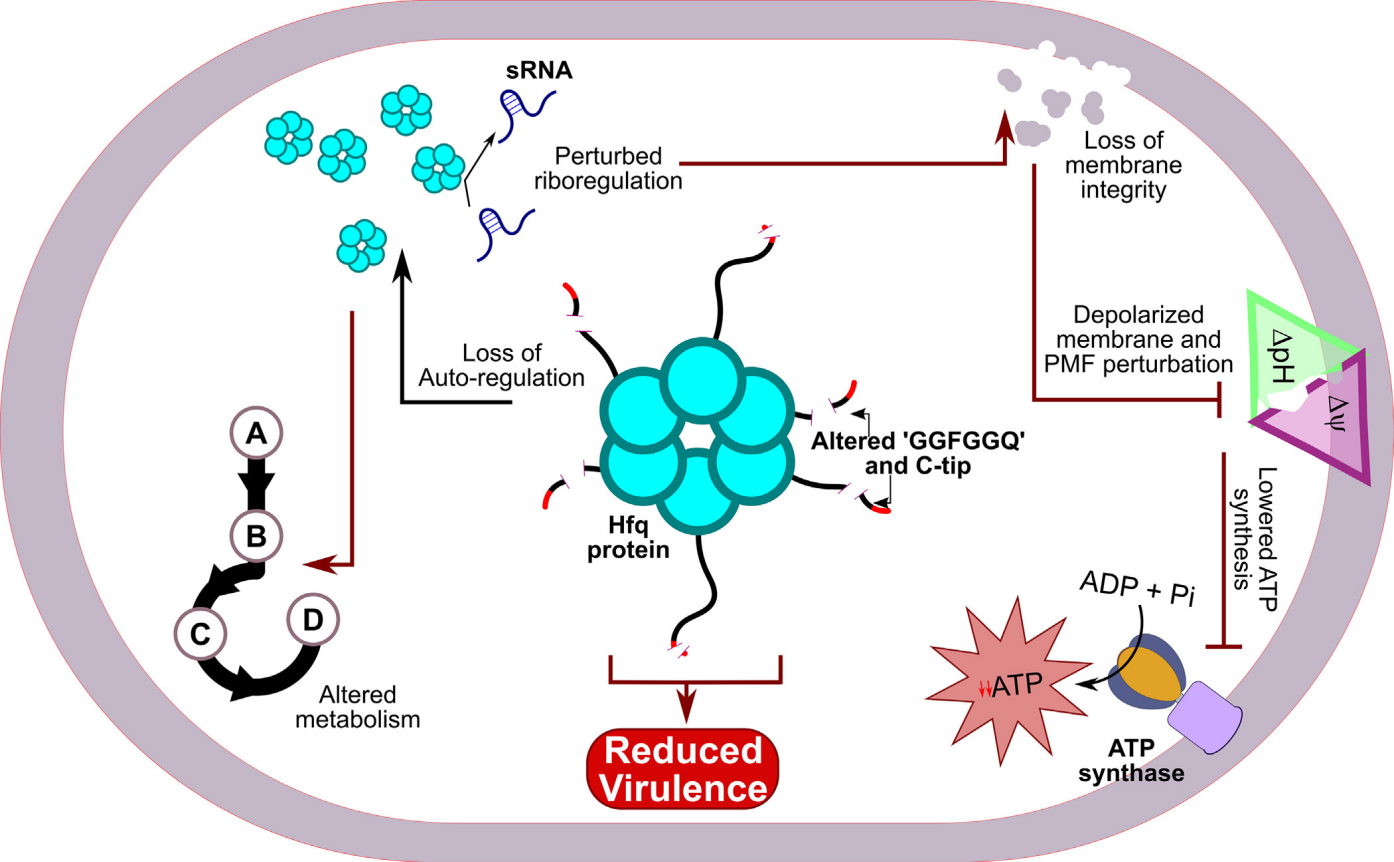
**A schematic overview.** The pathophysiological role of *A. baumannii* Hfq with its redundant ‘GGFGGQ’ repeats and acidic C-tip in conferring fitness and augmenting virulence of this pathogen. Our study demonstrates that the alteration of the C-terminal domain (CTD) composition, particularly changes in the ‘GGFGGQ’ repeats and the acidic C-terminal tip (C-tip), results in changes in RNA affinity of this protein. Inside the cell, this results in loss of membrane homeostasis, disruption of the proton motive force (PMF), and reduced intracellular ATP levels. Through these pleiotropic changes, the Hfq CTD variants impart an altered metabolic state with a concomitant loss of virulence. Hence, highlighting that *A. baumannii* Hfq protein with its intact CTD is necessary to maintain optimal pathophysiological fitness of this pathogen.

The RNA chaperoning activity of the homohexameric Hfq protein brings about interaction between an sRNA and its cognate mRNA partner through its Sm-core domain (7). However, a bacterial cell may have a menagerie of sRNAs being transcribed at any given point in time. Hence, the Hfq protein must prepare itself for its chaperoning activity after every round of sRNA–mRNA interaction. In *E. coli*, it was found that the Hfq turnover is mediated by its CTD, especially with the help of its acidic tip (20, 21). The presence of a highly conserved Hfq CTD in different strains and species of *Acinetobacter* (Fig. S2) led us to investigate whether changes in the ‘GGFGGQ’ repeats and C-tip of Hfq affects the pathophysiological fitness of this pathogen. In our assays, it was established that the *A. baumannii* Hfq variants exhibit altered binding affinity to an RNA probe depending upon the proximity and composition of the acidic C-tip. Although alterations of the ‘GGFGGQ’ repeats or the acidic tip do not completely render the Hfq inactive, it causes perturbations in its ability to interact with RNA molecules. Moreover, it was observed that the intact Hfq CTD, *that is*, the number of ‘GGFGGQ’ repeats and acidic amino acids of the C-tip, are implicated in optimally auto-regulating its own expression *in vivo* (Fig. 7). Our findings also implicate the ‘GGFGGQ’ repeats and C-tip of Hfq in maintaining membrane homeostasis, cellular PMF, and intracellular ATP levels (Fig. 7). These perturbations contribute towards an altered metabolism and reduced virulence (Fig. 7), thus pointing out the physiological importance of ‘GGFGGQ’ repeats along with the C-tip of *A. baumannii* Hfq. Several reports have highlighted that deletion of *hfq* results in a compromised growth phenotype (27). Perturbed growth resulting from *hfq* deletion is brought about by a wide array of cellular changes due to the pleiotropic nature of Hfq-mediated post-transcriptional regulation. Hfq is known to be involved in a noncoding arm of membrane homeostasis in pathogens like *E. coli* and *Salmonella* sp. (44, 45). In *E. coli*, loss of *hfq* results in a loss of outer membrane homeostasis with a concomitant increase in OMP levels (45, 52). In *Pseudomonas aeruginosa*, a taxonomically close genus of *A. baumannii*, deletion of *hfq* leads to overexpression of OMPs like *oprD* and *oprQ* (53). Loss of membrane integrity, in general, can lead to PMF disruption and reduced ATP synthesis (54). Similar observations were made in *Pseudomonas putida* Δ*hfq* strain where growth defect is accompanied by lowered intracellular ATP levels and reduced biofilm formation (55). From a previous study in *A. baumannii*, we had established that deletion of *hfq* or complete removal of its CTD along with acidic C-tip results in a loss of pathophysiological fitness (6). In addition, *A. baumannii* Δ*hfq* cells show an inability to optimally utilize different carbon sources, exhibit reduced biofilm formation, and are less virulent than the WT (6). These changes in pathophysiology can be attributed to perturbation of post-transcriptional regulation. Thus, our findings are in agreement with previous reports on the role of Hfq in different bacterial strains. Therefore, changes in the conserved *A. baumannii* Hfq CTD perturb its ability to optimally function within the cell.

The Hfq protein is known to be involved in a pleiotropic role, especially in conferring physiological fitness. Hence, we subjected *A. baumannii* strains harboring the Hfq mutants to different physiological assays. From these assays, it was found that the ‘GGFGGQ’ repeats, along with the acidic amino acid C-tip, are important in the proper functioning of Hfq within *A. baumannii* (Fig. 7). We have found that complete removal of the CTD (Hfq_66_) or reduction in the ‘GGFGGQ’ repeat length while keeping the C-tip intact, that is, Hfq_NG0_ and Hfq_NG1_, had a more pronounced effect than alterations in the C-tip composition, that is, Hfq_AbEc_ and Hfq_T0_. Taken together, our observations indicate that the pattern of growth defect observed with all the mutants in LB broth (Fig. 3*A*) is accentuated in different stress conditions, that is, nutrient limitation, stress susceptibility, biofilm formation, and virulence. From our findings, it is evident that the ‘GGFGGQ’ amino acid redundancy of Hfq CTD is not translated to its functions. The high degree of conservation of Hfq locus among clinical strains of *A. baumannii* and other *Acinetobacter* species (Figs. S2 and S3*C*) further supports our observations. This result is of even more importance as alterations in the CTD of Hfq result in defects in stress tolerance, carbon source utilization, and biofilm formation. These traits enable *A. baumannii* to survive in its soil microenvironments and also help it establish successful infections. We found that these traits are translated to the ability of the ‘GGFGGQ’ mutants to establish lung infection. These strains were severely attenuated in their ability to colonize the lung and spread to other host organs like the spleen, kidney, and liver. However, we observed an anomaly with the Hfq_NG1_ mutant, as in all our experiments, this mutant exhibited similar fitness perturbations as the *hfq* deletion (*Δhfq*) mutant despite possessing the Sm-core along with a part of the disordered CTD and the native C-tip. We believe that this could be due to the inability of Hfq_NG1_ to properly interact with its RNA target, as was established in our RNA affinity (Fig. 2*C*) and auto-regulation assays (Fig. 2). However, further studies must be carried out with this mutant (Hfq_NG1_) to validate this hypothesis. Overall, these observations are critical in establishing the importance of the ‘GGFGGQ’ repeats in proper functioning of the *A. baumannii* Hfq.

We included the C-tip mutants in our study to explicitly understand whether changes in the local acidic amino acid composition render the Hfq nonfunctional inside the cell. As the C-tip is involved in the regulation of Hfq turnover in *E. coli* (21), we substituted the tip acidic residues with that of *E. coli,* that is. four acidic amino acids instead of the seven of *A. baumannii* Hfq (Hfq_AbEc_) (Figs. S2 and 2*A*). The other mutant with no acidic amino acids at the tip was generated to replicate the *Clostridium* sp. Hfq that has no acidic amino acid on its tip (Hfq_T0_) (Figs. S2 and 2*A*). In both cases, we found that alterations in the C-tip do reduce the pathophysiological fitness of this pathogen, albeit not to the extent exhibited by ‘GGFGGQ’ alterations. We hypothesized that the long length of the CTD in *Acinetobacter* sp. and other members of the Moraxellaceae family is accompanied by an increase in the local acidic amino acid residues at the C-tip. This hypothesis is supported by the fact that there seems to exist a direct correlation between the length of the CTD and the number of acidic amino acids at the C-tip (Fig. S2). Hence, our C-tip mutants, due to the loss of the acidic residues, were unable to optimally carry out Hfq turnover, leading to pathophysiological defects.

It is of interest to note that Hfq is known to be involved in the regulation of catabolism in *Pseudomonas* sp. (56). In *Pseudomonas* sp., Hfq, along with another protein called CRC, establishes carbon catabolite repression or CCR (56, 57). Such Hfq–protein interactions are not unique, as Hfq is also known to interact with other important proteins within a bacterial cell, like the degradosome complex and polyA polymerase (58, 59, 60). Other than directly interacting with proteins, Hfq indirectly contribute towards activation of various sigma factors like σ^E^ (45) and post-transcriptionally regulate others like σ^S^ (61, 62). Additionally, Hfq is known to form CTD-mediated liquid-liquid phase-separated bodies (63, 64, 65). A recent finding reported that Hfq can colocalize with RNaseE and other components of the degradosome complex in a liquid-liquid phase-separated body termed as the H-body (66). Hence, the length of the Hfq CTD has far-reaching implications beyond its RNA chaperoning activity. Through our studies, we have highlighted that complementing the *hfq* deletion mutant with Hfq variants harboring altered CTD cannot fully restore the function of full length Hfq. This observation can be attributed to the fact that the pleiotropic effect of Hfq is brought about by a plethora of cellular circuits involving sRNA and other regulatory proteins, transcription factors, and two component systems. Hence, future investigation into how these alterations affect *A. baumannii* Hfq in its ability to interact with other cellular proteins holds immense potential.

Our understanding of the role of the unique CTD of *A. baumannii* Hfq does not provide conclusive evidence towards the endogenous role of this protein in establishing a post-transcriptional network. Although there has been some evidence of Hfq-influenced, RNaseE-mediated degradation of sRNA targets in *A. baumannii* (67), endogenous roles are yet to be deciphered. One way to do the same is to screen for Hfq-bound sRNA-mRNA partners using various pull-down and deep sequencing strategies (68, 69, 70, 71). Amongst these, RIL-seq (RNA interaction by ligation and sequencing) and CLASH (UV cross-linking, ligation and sequencing of hybrids) have been the most frequently implemented techniques in recent years (69, 70, 72, 73, 74). These techniques generally involve FLAG-tagging the Hfq protein at its C-terminal end, allowing the pull-down experiments to be carried out using an anti-FLAG antibody (69, 71). However, as our data suggests, alterations in the CTD also influence the overall physiological functioning of Hfq inside the cell. Hence, FLAG tagging the CTD may influence the overall data generated by these studies and may miss out on novel physiologically important interactions. Results from our study will help in guiding the development of better strategies to study the endogenous role of *A. baumannii* Hfq and its CTD. Furthermore, as the CTD of Hfq is implicated in roles beyond RNA chaperoning in different bacterial strains, future studies with our mutants may uncover novel cellular mechanisms that aid this pathogen survive and infect its host.

## Experimental procedures

### Strains, plasmids, and culture conditions

The strains used in this study are listed in Table S1. For culturing bacteria, Luria Bertini broth (Himedia) and 1X M9 minimal salts (Himedia) supplemented with 10 mM of indicated carbon source (amino acids, carbohydrates, and organic acids). All carbon sources were dissolved in milliQ water and filter sterilized using a 0.22 μM PVDF syringe filter (Millipore) (Tyr was first dissolved at 55 °C). All the carbon source were freshly prepared before each experiment. The nutrients were half diluted using sterile 2X M9 salts which were then inoculated with strains under investigation, a detailed methodology for the same is discussed below. All the amino acid sources were procured from TCI, and the carbohydrates and organic acids were procured from Himedia. For all the stress condition assays, sterile LB broth was supplemented with iron limitation (200 μM 2,2-dipyridyl) (Sigma), oxidative stress (200 μM methyl viologen) (Sigma), osmotic stress (by additionally supplementing LB broth [Himedia] with 1% or 171.1 mM, 2% or 342.3 mM, and 3% or 513.4 mM NaCl [Himedia], w/v), and pH stress (pH 2, 4, 5, and 8.5, adjusted with HCl [Himedia]). All the cultures were grown at 37 °C with 180 rpm unless mentioned otherwise. All the growth profiling was carried out on 96-well plates in a multimode plate reader (Synergy H1) at 37 °C with shaking. LB was used for culturing bacteria and was supplemented with 30 μg/ml chloramphenicol, 100 μg/ml (*E. coli*) and 200 μg/ml (*A. baumannii*) ampicillin, 20 μg/ml (*E. coli*) and 40 μg/ml (*A. baumannii*), and 50 μg/ml (*E. coli*) or 15 μg/ml (*A. baumannii*) kanamycin, for maintaining plasmids whenever required. The plasmids and oligonucleotide primers used in this study are listed in Tables S2 and S3, respectively.

### Multimode plate reader–based growth assessment

#### Inoculum preparation

In brief, we inoculated *A. baumannii* WT and mutant strains from single colony into respective culture tubes in 5 ml LB broth. Next, we incubated these culture at 37 °C with shaking at 180 rpm for 18 h overnight. The overnight cultures were 1000 times diluted in fresh 5 ml LB broth and incubated under the same conditions. The cultures were allowed to reach A_600_ = 0.5 to 0.7, and subsequently, the A_600_ was adjusted to 0.4. From this, 0.4 A_600_ culture 1 ml aliquot was taken in a 1.5 ml MCT and washed twice using 1 ml 2X sterile M9 salts (without any carbon source) by centrifugation at 5000 rpm for 5 min and finally resuspending in the same solvent. This resuspension will be subsequently diluted and used as inoculum for the 96-well growth-kinetic plate experiments.

#### Single-nutrient 96-well growth kinetics

To prepare the 96-well plates for the growth curve assays, 100 μl of sterile milliQ water supplemented with twice the amount of the desired concentration of nutrient (*i.e.*, the final concentration of nutrient was 10 mM, we dispensed 100 μl of 20 mM nutrient into the respective wells). Next, in a sterile reagent reservoir, a required volume of 2X M9 salts without any carbon source was added. To this, the 0.4 A_600_ cell resuspension was added (final dilution: 0.2% v/v). Next, 100 μl of this culture inoculum was dispensed and mixed with a multichannel pipette into wells marked for the different strains used in this study. Upon adding the inoculum mix, the nutrient concentration, inoculum load, and 2X M9 salts are half diluted to 10 mM, 0.1%, and 1X M9 salts, respectively. The plate is then kept in a multimode plate reader with lid (Synergy H1, Biotek) pre-set at 37 °C and A_600_ is measured at desired time intervals till 12 h with shaking. The A_600_ versus Time is plotted as a XY curve in GraphPad (Prism 8) software.

#### Bioinformatic analysis

The computational analysis has been detailed in supporting information section S1.

### Recombinant DNA and genomic complementation procedures

The WT Hfq and Hfq_66_ proteins with stop codon were cloned into a pET-28 vector between NcoI and XhoI sites on the plasmid and PscI (isocaudomer of NcoI) and XhoI sites on the insert. The other Hfq CTD variants except for Hfq_AbEc_ were generated by infusion cloning (Takara) of inverse PCR amplification products of WT hfq gene cloned in pET28 (pET-WT) (Fig. S4*B*). The Hfq_AbEc_ was constructed by ligating two PCR products, one being the inverse PCR product of pET-WT without the C-tip and the other being the C-tip of *E. coli* Hfq with its stop codon. Both the PCR products had homologous ends corresponding to the other to allow for a seamless infusion cloning of the two (Fig. S4*B*). These plasmids were transformed into *E. coli* BL21(DE3) cells and subsequently overexpressed as tag-free Hfq variants. The variants were purified using a nickel-NTA column (Cytiva) on a FPLC system (AKTA Pure) as described below. Genomic complementation of CTD variants was created using a homologous recombination–based approach as described by Dubey et, al (23). In brief, a nucleic acid sequence of Hfq CTD variants was amplified from the respective clones in pET-28 vectors. The PCR products were subsequently cloned in a pUC18 vector between the upstream 500 bp region (US500) of *hfq* on its 5′-end and an apramycin-FRT cassette followed by downstream 500 bp region of *hfq* on its 3′-end. A PCR amplicon from these clones were used for genomic complementation of the *hfq* variant gene into the native *hfq* genomic locus of an *A. baumannii Δhfq* strain. All of the other methods pertaining to recombinant DNA; genomic complementation and plasmid complementation under native promoter are described in supporting information section S2.

### Purification of the Hfq variant proteins

The Hfq variants cloned into pET-28 vector were transformed into *E. coli* BL21(DE3) cells. The cells were induced with 0.25 mM IPTG (Himedia) and incubated at 18 °C for 16-18h. The cells were washed in 1X PBS and lysed using a lysis buffer and purified using a His Trap FF column (5 ml) (Cytiva) in the AKTA Pure system (75). The detailed methodology is mentioned in supporting information section S3.

### Probing for Hfq variants RNA-binding affinity

Fluorescence anisotropy (FA) was measured with a BioTek Synergy H1 (Agilent) using 96-well plates (Thermo Fisher Scientific, flat-bottom-black) in triplicates and a final reaction volume of 100 μl per well. All reactions were performed in TNK buffer (10 mM Tris·HCl, pH 7.5, 50 mM NaCl, 50 mM KCl). Each well had 50 nM FAM-labeled D16 sRNA probe and serial dilution of each protein ranges from 50 nM to higher until the saturation of the fluorescent anisotropy signal was reached. Bovine serum albumin with different concentrations with the same parameters was used as a negative control. Background FA was recorded with the same parameters without the addition of proteins. FA signals of D16FAM were taken every 20 s for >200 s with gentle shaking at 30 °C, applying polarization filters with an excitation wavelength of 485 nm and an emission wavelength of 528 nm. For *K*_*d*_ determination, the FA data were analyzed by following equation [Equation (1)] (76) using KaleidaGraph software.

(Eq. 1) $${A=A}_{0}+\left(A_{\max}-A_{0}\right)*\frac{\left(\left[P_{T}\right]+\left[S_{T}\right]+K_{d}\right)-\sqrt{{\left(\left[P_{T}\right]+\left[S_{T}\right]+K_{d}\right)}^{2}-4\left[P_{T}\right]\left[S_{T}\right]}}{2\left[S_{T}\right]}$$

where A = Experimental fluorescence anisotropy, A0 = the fluorescence anisotropy of the free D16-FAM sRNA, Amax = the fluorescence anisotropy for the fully bound D16-FAM sRNA, [PT] = Varying concentration of Hfq protein (nM), and [ST] = the total added concentration of D16-FAM sRNA (=50 nM)

### Auto-regulation assay

Plasmid pR131 contains an *hfq-lacZ* translational fusion. The plasmid was transformed into *E. coli Δhfq*. The plasmids (pWHN678) carrying the complementing variants of *hfq* were individually cotransformed along with pR131. The cells were grown until exponential phase, and overexpression of Hfq-LacZ was induced by the addition of 2 mM IPTG for 30 min. Hundred microliters of culture was withdrawn, and its absorbance at 600 nm was measured using a flat-bottom 96-well plate (Corning). The cells were washed twice in 1X PBS. The washed cells were resuspended in 1 ml 1X PBS (with no carbon source). To these, a final concentration of 1 mM chlorophenol red-β-D-galactopyranoside was added (Charlotte Jendresen, 2017). After 8 h of incubation at 30 °C, 100 μl was aliquoted and A_570_ was measured using a flat-bottom 96-well plate (Corning). The β-galactosidase activity of chlorophenol red-β-D-galactopyranoside–based assay was expressed in terms of Miller units using Equation (2).:

(Eq. 2) $$\beta -\text{Gal}\;\text{activity}=\frac{1000\times A_{570}}{Vol\;\left(mL\right)\times time\;\left(\text{minutes}\right)\times A_{600}}$$

### Biofilm formation assay

*In vitro* biofilm formation was studied by sub-culturing 0.6 A_600_ *A. baumannii* cells in 1 ml LB broth in a 12-well tissue culture grade plate (Tarsons). Separate plates were inoculated for 48 h and 72 h at 30 °C, while keeping them covered with aluminium foil under static conditions. After incubation, the cell suspension was collected and its A_600_ was determined. The wells were washed with copious amounts of sterile water and allowed to dry. One milliliter of 0.1% freshly prepared crystal violet solution was added to each well and incubated at room temperature for 30 min. The wells were washed again with copious amounts of sterile water. The stain was dissolved in 1 ml of 30% acetic acid and A_575_ was recorded. The ratio of A_575_ and A_600_ was determined for each strain.

### Assays for measuring ATP levels, membrane polarization, and outer membrane permeability

All the different strains of WT and Hfq mutants of *A. baumannii* were subjected to intracellular ATP quantification using BacTiter-Glo kit (Promega) according to the manufacturer’s protocol. The WT cells treated with 5 μM CCCP (Sigma) was used as a positive control. To determine the membrane polarization of WT and mutant strains, the *A. baumannii* cells were stained with DiBAC4 (Sigma) dye that exhibit enhanced fluorescence upon encountering a depolarized membrane. Finally, N-phenyl-1-naphthylamine (Sigma) was used to investigate the degree of outer membrane permeability. A 0.5 × MIC concentration of polymyxin B–treated WT cells was used as a positive control. All the reads were measured using a multimode plate reader. A detailed protocol for each of these assays is described in the supporting information section S3.

### Quantification of RNA transcripts using quantitative real time-PCR

*A. baumannii* cells were incubated at 37 °C in 5 ml in sterile borosilicate culture tubes with shaking at 180 RPM till A_600_ = 0.6. The cells were then washed twice in sterile 1X PBS. After washing the cell pellet with 1X PBS, RNA was extracted from the bacterial cells using the classic phenol-chloroform method. Complementary DNA synthesis was performed using the PrimeScript 1st strand complementary DNA Synthesis Kit (TakaRa) according to the manufacturer’s instructions. Amplifications were achieved using a 3-step program on a QuantStudio 5 system (Thermo Fisher Scientific). Transcript abundance was calculated using the ΔΔC_T_ method and normalized by the 16s gene.

### Scanning electron microscopy analysis

Overnight cultures of *A. baumannii* cells were incubated at 37 °C in 5 ml in sterile borosilicate culture tubes with shaking at 180 RPM till A_600_ = 0.6. The cells were then washed and fixed resuspended in sterile 1X PBS. Overnight fixation of the cells was carried out at 4 °C using a glutaraldehyde-formaldehyde mix (2.5% and 2%, v/v, respectively) added to the cell suspension. The cells were dehydrated in an ethanol gradient and visualized in a scanning electron microscope (FESEM-Zeiss Ultra Plus).

### *A. baumannii* murine pneumonia model to assess virulence

All animal experiments under protocol BT/IAEC/2018/07 were reviewed and approved by the Institute Animal Ethics Committee of the Indian Institute of Technology Roorkee. Adult (6–8 week old) age-matched female BALB/c mice (n = 6 for each group) were procured. The mice were quarantined for 1 week with free access to food and water along with a 12-h day-night cycle. On the −4 and −1 day of the experiment, the mice were immunocompromised *via* intraperitoneal injection of cyclophosphamide (150 mg/Kg body wt.) (TCI). On day 0, the mice were anaesthetized with a 1:1 dose of ketamine (75 mg/Kg body wt; IP) (Themis Medicare) and xylazine (16 mg/Kg body wt; IP) (Indian Immunologicals). Subsequently, the mice were infected intranasally with *A. baumannii*, and CFU was enumerated as previously described. Briefly, the mice were anaesthetized and infected intranasally with 20 μl of inoculum containing 2 × 10ˆ9 CFU of the indicated strains. Mice were euthanized 36 h post-infection, and the lungs, liver, kidneys, and spleen were harvested, immediately transferred on ice, and washed with ice-cold 1X sterile PBS. The harvested organs were homogenized to enumerate the bacterial burden by serially diluting and plating on Leeds *Acinetobacter* agar plates (Himedia). To perform tissue histology, a single lobe of the lung was removed and imaged after H&E staining by Kaushik Pathology Clinic.

### Statistical analysis

Statistical analyses were carried out using Prism 8 software (GraphPad). One-way ANOVA with Tukey’s multiple-comparison *post hoc* test was used for comparisons between three or more conditions. *p*-value was ∗, *p* ≤ 0.05; ∗∗, *p* ≤ 0.001; ∗∗∗∗, *p* ≤ 0.0001; ns, nonsignificant.

## Data availability

All data supporting the findings of this study are available from the corresponding author upon request.

## Supporting information

This article contains supporting information (6, 14, 21, 23, 24, 25, 75, 77).

## Conflicts of interest

The authors declare that they have no conflicts of interests with the contents of this article.
