# Supplementary material for: ‘GGFGGQ’ repeats in Hfq of Acinetobacter baumannii are essential for nutrient utilization and virulence
Source: J Biol Chem. 2024 Oct 17;300(12):107895. doi: 10.1016/j.jbc.2024.107895 (PMC11617691; doi:10.1016/j.jbc.2024.107895)
Supplement: Supporting information [file mmc1.docx]

**‘GGFGGQ’ repeats in Hfq of *Acinetobacter baumannii* are essential for nutrient utilization and virulence**

Abhiroop Sett, Pulak Kumar Maiti, Kritika Garg, Arsalan Hussain, Snehlata Saini, Shivam Pandey, Ranjana Pathania.

**List of materials included:**

**Supporting experimental procedures……………………………………..…........S2**

S1. Bioinformatic analysis……………………………………………………..……….S2

S2. Recombinant DNA procedures……………………………………………………S2

S3. Purification of Hfq and variants ………………………..……………………..…..S3

S4. Cellular energetics assays ……………………………………………….…….…S3

**Supporting Tables……………………….…...…………………………………….....S4**

Table S1………………………………………………………………………………….S4

Table S2……………………………………………………………………………….…S6

Table S3………………………………………………………………………………….S7

**Supporting Figures …………..………………………….………………………......S10**

Figure S1……………………………………………………………………….……….S10

Figure S2……………………………………………………………………….……….S11

Figure S3……………………………………………………………………….……….S12

Figure S4…………………………………………………………………….………….S13

Figure S5……………………………………………………………………………......S14

Figure S6………………………………………………………………………………...S15

Figure S7…………………………………………………………………………..........S16

Figure S8……………………………………………………………………………......S18

Figure S9………………………………………………………………………….........S20

**Supporting references……………………………………………………………….S21**

**S1. Bioinformatic analysis**

**Prediction of *A. baumannii* Hfq structure using Alphafold2**

The wild type *A. baumannii* Hfq protein sequence was obtained from Uniprot. The protein structure was predicted with Alphafold2 (<https://colab.research.google.com/github/sokrypton/ColabFold/blob/main/AlphaFold2.ipynb>) using default parameters. The plDDT score of the Sm-core was very high (>90) while that of the intrinsically disordered CTD was very low (<50). The predicted sequence .pdb file was colour coded using UCSF Chimera

**Multiple sequence alignment**

All the sequences were retrieved from NCBI genome database (<https://www.ncbi.nlm.nih.gov/datasets/genome/>). Multiple sequence analysis was performed using EMBL-EBI muscle alignment tool (<https://www.ebi.ac.uk/jdispatcher/msa/muscle?stype=protein>). The alignments were visualized using Jalview for windows 64-bit. The colour scheme is according to the Clustal X Default colouring scheme.

**S2. Recombinant DNA procedures**

**S2.1. Genomic complementation of *hfq* variants**

Sequential cloning of 5’-upstream (US) 500 bp region of *hfq*, Apramycin-FRT cassette, and 3’ downstream (DS) 500bp region of *hfq* was done in a pUC18 plasmid. Next, the genetic sequence of the *hfq* variants cloned into the pET-28 vector were amplified using Hfq-CDS_KI-F and Hfq-CDS_KI-R for all the variants. These amplicons were cloned in between the 5’-upstream 500 bp region of *hfq*, Apramycin-FRT cassette in a seamless manner using the infusion cloning strategy (Takara). Next, 150bp internal primers corresponding to the US and DS 150bp regions were used to amplify the genomic constructs to be used for integrating the Hfq mutant into the genome of *A. baumannii Δhfq* strain. The chimeric PCR product was concentrated to 5mg and transformed into *A. baumannii* ATCC17978 *Δhfq* electrocompetent cells with pAT02 (induced by 5mM isopropyl-β-_D_-thiogalactopyranoside [IPTG]). The transformants were screened for positive knock-in using the primers Hfq CDS FP and Hfq CDS RP, keeping genomic DNA from WT and the *Δhfq* strains as controls. The apramycin cassette was removed from the positive transformants by using pAT03 expressing FLP recombinase. The variants were confirmed using PCR and the amplicons were resolved on a 1% agarose gel.

**S2.2. Plasmid borne complementation of *hfq* with its native promoter and terminator**

Genomic DNA from *A. baumannii* strains with Hfq protein variants integrated into them was used as a template for PCR amplification of the *hfq* variants along with its native promoter and terminator regions. The primers used were the same that was used in our previous study (1). OligoRPT41 and OligoRPT42. The PCR products were cloned in between BamHI sites on a pWHN678 plasmid and transformed into *E. coli* DH5α chemical competent cell by heat shock method. Transformants were selected by spreading the *E. coli* cells post-recovery on LB agar plates supplemented with 30mg/L chloramphenicol.

**S3. Purification of Hfq and variants**

The proteins were expressed and purified as previously described with some modifications (2). In brief, *E. coli* BL21-DE3 harbouring pET-28 plasmids expressing Hfq wild type, Hfq66, HfqNG0, HfqNG1, HfqT0, and HfqAbEc were grown in 1 lt of LB at 37 °C to an A_600_ of 0.5–0.6. Cells were then treated with 0.25 mM IPTG and allowed to grow for 16–18 hours at 18°C. The pellets were washed once in 1X PBS and dissolved in 50 ml lysis buffer [50 mM Tris (pH 7.5), 1. M NaCl, 1 M urea, 1 mM β-mercaptoethanol, 5% glycerol, along with 50 µl each of PMSF, DNase I (0.1 mg/ml) and 1 M MgCl2. Following vortex, homogenization, and lysis using French press, the supernatant was collected by centrifugation. Again, the collected supernatant was ultracentrifuged for 30 min at 1,00,000 rcf. The clear supernatant was heated at 95 °C for 15 min, clarified by centrifugation, and treated with 30 µg/ml of RNaseA for 1 h at 37 °C. After RNaseA treatment, the supernatant was collected by high-speed centrifugation. The cell lysates were filtered through a 0.2 µm membrane filer and loaded onto His Trap FF column (5 ml) (Cytiva) in the AKTA Pure. Before loading the samples, the column was equilibrated with washing buffer [50 mM Tris (pH 7.5), 1 M NaCl (pH 7.5), 1 mM β-mercaptoethanol] and proteins were binding to the column with a flow rate of 1ml/min. After binding of proteins, the column was washed for another 10 min with the same buffer, and finally, proteins were eluted with elution buffer [50 mM Tris (pH 7.5), 1 M NaCl (pH 7.5), 500 mM imidazole, 1 mM β-mercaptoethanol, l5% glycerol]. The 10 kDa spin column was used to concentrate the fractions, and the Hfq proteins were gathered in a snakeskin dialysis bag and exposed to overnight dialysis in Hfq buffer (50 mM Tris (pH 7.5), 50 mM NH4Cl, 1 mM EDTA, and 10% glycerol). All the purified proteins were resolved on a 15% SDS PAGE gel

**S4. Cellular energetics assays**

**S4.1. Assay for determining intracellular ATP**

BacTiter-Glo kit (Promega) was used to determine the intracellular ATP levels as per the manufacturer’s protocol. In brief, the WT and mutant strains of *A. baumannii* were sub-cultured and allowed to grow till A_600_ of 0.6 at 37 ⁰C with shaking (180 RPM) in LB broth. 1 mL aliquots of the cells were washed twice in sterile 1X PBS, and one of the WT cell aliquot was subjected to 5µM CCCP treatment for 30 minutes. The cells were then added to 96-well round bottom white well plate (Corning) and to it the BacTiter-Glo reagent was added and mixed. The luminescence was measured using a multimode plate reader (Synergy H1). The luminescence was normalized by first subtracting ATP levels in the spent media and then normalizing the values by dividing by the absorbance read at 600 nm.

**S4.2. Assay for Outer Membrane Permeability**

N-phenyl-1-naphthylamine (NPN) (Sigma) was used to assess the outer membrane permeability in the presence of compounds. NPN binds to the outer leaflet of the cell membrane and fluoresces when in a hydrophobic environment. Overnight-grown cultures of *A. baumannii* ATCC17978 and the genome-complemented Hfq mutants were sub-cultured in fresh LB medium and incubated at 37°C and 180 RPM until an A_600_ of 0.6 was reached. The cells were then washed and resuspended in 5 mM HEPES buffer to an A_600_ of 0.3. As a control, an 0.3 A_600_ 1mL-aliquot of the WT cells were incubated with 0.125 mg/L or 0.5XMIC of polymyxin B (Sigma) for 30 mins. Next, the cells were washed and resuspended in sterile 1XPBS and 100 µL of each variants and positive control cells were aliquoted into an opaque plate (Thermo scientific). NPN (10 μM) was added to the wells, and fluorescence was immediately measured at excitation/emission wavelengths of 350/420 nm using a multimode plate reader (Synergy H1). Relative Fluorescence Units (RFU) were calculated by dividing the fluorescence values obtained by the absorbance at 600 nm. The relative change in fluorescence compared to the WT *A. baumannii* cells and were plotted.

**S4.3. Assay for measurement of membrane potential**

Exponential phase *A. baumannii* ATCC17978 cells and genome-complemented Hfq mutants grown in LB were washed and diluted to an A_600_ of ~0.3 in 1X PBS. A 1 mL aliquot of the WT cells was treated with 5µM CCCP (Sigma) as a positive control, which was washed again after 30 mins and cells were resuspended in 1X PBS. Next, 1 mL aliquots of each of these cells were incubated with DiBAC4 at 10 μM for 30 minutes, followed by three washing steps with 1X PBS. The cells were then added to the wells of a 96-well opaque plate (Thermo) and fluorescence was recorded at an excitation/emission wavelength of 490/516 nm using a multimode plate reader (Synergy H1). RFU was calculated by normalizing against A_600_, and the relative change in fluorescence in the Hfq mutants compared to the WT was plotted.

**Supporting Tables**

**Table S1.** List of bacterial strains used in this study

| Sl. No. | Strain | Relevant Characteristics | Source/ reference |
| --- | --- | --- | --- |
| 1 | *Acinetobacter baumannii* ATCC17978 | Wild type (WT) *A. baumannii* strain | Purchased from ATCC |
| 2 | *E. coli* Bl21 (DE3) | *hsdS gal* (*cIts*857 *ind*1 *Sam*7 *nin*5 *lacUV5*-T7 gene *1*) | Invitrogen, USA |
| 3. | *E. coli* DH5α | *supE44 hsdR17 recA1 endA1 gyrA96 thi-1 relA1* | Invitrogen, USA |
| 4. | *E. coli* HST08 | F-, endA1, supE44, thi-1, recA1, relA1, gyrA96, phoA, Φ80d lacZΔ M15, Δ(lacZYA-argF) U169, Δ(mrr-hsdRMS-mcrBC), ΔmcrA, λ- | Takara, Japan |
| 5. | *E. coli Δhfq* | Deletion mutant of *hfq* in *E. coli* BW25113 | (3) |
| 6. | RPTC1 | *A. baumannii* clinical isolate | (4) |
| 7. | RPTC 2 | *A. baumannii* clinical isolate | (4) |
| 8. | RPTC3 | *A. baumannii* clinical isolate | (4) |
| 9. | RPTC5 | *A. baumannii* clinical isolate | (4) |
| 10. | RPTC6 | *A. baumannii* clinical isolate | (4) |
| 11. | RPTC9 | *A. baumannii* clinical isolate | (4) |
| 12. | RPTC11 | *A. baumannii* clinical isolate | (4) |
| 13. | RPTC12 | *A. baumannii* clinical isolate | (4) |
| 14. | RPTC14 | *A. baumannii* clinical isolate | (4) |
| 15. | RPTC15 | *A. baumannii* clinical isolate | (4) |
| 16. | RPTC16 | *A. baumannii* clinical isolate | (4) |
| 17. | NKN185 | *Acinetobacter baylyi* | Gift from Prof. Naveen K. Navani |
| 18. | NKN186 | *Acinetobacter calcoaceticus* | Gift from Prof. Naveen K. Navani |
| 19. | NKN187 | *Acinetobacter Johnsonii* | Gift from Prof. Naveen K. Navani |
| 20. | RPT 233 | *Acinetobacter baumannii* ATCC17978 *Δhfq* | (1) |
| 21. | RPT614 | *Acinetobacter baumannii* ATCC17978 with Hfq_66_ on the genome. | This study |
| 22. | RPT615 | *Acinetobacter baumannii* ATCC17978 with Hfq_NG0_ on the genome. | This study |
| 22. | RPT616 | *Acinetobacter baumannii* ATCC17978 with Hfq_NG1_ on the genome. | This study |
| 23. | RPT617 | *Acinetobacter baumannii* ATCC17978 with Hfq_AbEc_ on the genome. | This study |
| 24. | RPT618 | *Acinetobacter baumannii* ATCC17978 with Hfq_T0_ on the genome. | This study |
| 25. | RPT258 | *E. coli Δhfq* carrying pWHN678 | (1) |
| 26. | RPT260 | *E. coli Δhfq* carrying pWHN678-Hfq_66_ | (1) |
| 27. | RPT653 | *E. coli Δhfq* carrying pWHN678-Hfq_AbEc_ | This study |
| 28. | RPT654 | *E. coli Δhfq* carrying pWHN678-Hfq_T0_ | This study |
| 29. | RPT655 | *E. coli Δhfq* carrying pWHN678-Hfq_NG0_ | This study |
| 30. | RPT656 | *E. coli Δhfq* carrying pWHN678-Hfq_NG1_ | This study |
| 31. | RPT380 | *E. coli* DH5α carrying pET-WT | This study |
| 32. | RPT161 | *E. coli* DH5α carrying pET-Hfq66 | This study |
| 33. | RPT518 | *E. coli* DH5α carrying pET-HfqNG0 | This study |
| 34. | RPT497 | *E. coli* DH5α carrying pET-HfqNG1 | This study |
| 35. | RPT602 | *E. coli* DH5α carrying pET-HfqAbEc | This study |
| 36. | RPT662 | *E. coli* DH5α carrying pET-HfqT0 | This study |

**Table S2.** List of plasmids used in this study

| Sl. No. | Plasmid name | Relevant Characteristics | Source/ reference |
| --- | --- | --- | --- |
| 1 | pET-28 | Vector for expression of his-tagged recombinant proteins in *E. coli. kan^r^* | Novagen |
| 2 | pET-WT | *A. baumannii* WT Hfq cloned in pET-28 with its stop codon. *kan^r^* | This study |
| 3. | pET-Hfq66 | *A. baumannii* Hfq_66_ cloned in pET-28 with its stop codon*. kan^r^* | This study |
| 4. | pET-HfqNG0 | *A. baumannii* Hfq_NG0_ cloned in pET-28 with its stop codon*. kan^r^* | This study |
| 5. | pET-HfqNG1 | *A. baumannii* Hfq_NG1_ cloned in pET-28 with its stop codon*. kan^r^* | This study |
| 6. | pET-HfqAbEc | *A. baumannii* Hfq_AbEc_ cloned in pET-28 with its stop codon*. kan^r^* | This study |
| 7. | pET-HfqT0 | *A. baumannii* Hfq_T0_ cloned in pET-28 with its stop codon*. kan^r^* | This study |
| 8. | pWHN678 | Plasmid for cloning in *A. baumannii*. *chl^r^* | (5) |
| 9. | pRPT16 | *Hfq_66_* cloned in pWHN678; *chl^r^* | (1) |
| 10. | pWHN-NG0 | *hfq_NG0_* cloned in pWHN678; *chl^r^* | This study |
| 11. | pWHN-NG1 | *hfq_NG1_* cloned in pWHN678; *chl^r^* | This study |
| 12. | pWHN-AbEc | *Hfq_AbEc_* cloned in pWHN678; *chl^r^* | This study |
| 13. | pWHN-T0 | *Hfq_T0_* cloned in pWHN678; *chl^r^* | This study |
| 14. | pAT02 | Plasmid expressing *A. baumannii* RecT homolog. *amp^r^* | (6) |
| 15. | pAT03 | Plasmid expressing FLP recombinase enzyme (flippase) for expression in *A. baumannii*. *amp^r^* | (6) |
| 16. | pR131hfq | Plasmid with 131 nts of *hfq* ORF fused in frame with *lacZ* under *lac* promoter. *amp^r^* | (7) |
| 17. | pMDIAI | Plasmid containing Apramycin resistance cassette between FRT sites | Addgene, USA |
| 18. | pUC18 | pUC18 vector for cloning *hfq* knock-in constructs. *amp^r^* | Addgene, USA |
| 19. | pUC-Hfq-homolog | pUC18 harbouring 5’-upstream (US) 500 bp region of *hfq*, Apramycin-FRT cassette, and 3’ downstream (DS) 500bp region of *A. baumannii hfq. apr^r^* | This study |
| 20. | pUC-WT-KI | pUC18 harbouring 5’-upstream (US) 500 bp region of *hfq*, WT *hfq* CDS, Apramycin-FRT cassette, and 3’ downstream (DS) 500bp region of *A. baumannii hfq. apr^r^* | This study |
| 21. | pUC-66-KI | pUC18 harbouring 5’-upstream (US) 500 bp region of *hfq*, *hfq_66_* CDS, Apramycin-FRT cassette, and 3’ downstream (DS) 500bp region of *A. baumannii hfq. apr^r^* | This study |
| 22. | pUC-NG0-KI | pUC18 harbouring 5’-upstream (US) 500 bp region of *hfq*, *hfq_NG0_* CDS, Apramycin-FRT cassette, and 3’ downstream (DS) 500bp region of *A. baumannii hfq. apr^r^* | This study |
| 23. | pUC-NG0-KI | pUC18 harbouring 5’-upstream (US) 500 bp region of *hfq*, *hfq_NG1_* CDS, Apramycin-FRT cassette, and 3’ downstream (DS) 500bp region of *A. baumannii hfq. apr^r^* | This study |
| 24. | pUC-AbEc-KI | pUC18 harbouring 5’-upstream (US) 500 bp region of *hfq*, *hfq_AbEc_* CDS, Apramycin-FRT cassette, and 3’ downstream (DS) 500bp region of *A. baumannii hfq. apr^r^* | This study |
| 25. | pUC-T0-KI | pUC18 harbouring 5’-upstream (US) 500 bp region of *hfq*, *hfq_T0_* CDS, Apramycin-FRT cassette, and 3’ downstream (DS) 500bp region of *A. baumannii hfq* | This study |

**Table S3.** List of DNA and RNA oligos used in this study

| Sl. No. | Name of oligo | Sequence | Purpose |
| --- | --- | --- | --- |
| 1 | D16-FAM (RNA Oligo) | FAM-CGAAUUUUUUAAGUGC | Fluorescence anisotropy based titration assays (8) |
| 2 | 17978-16S-qRTF | ACTTTAAGCGAGGAGGAGGC | qRT-PCR primer for 16S transcript |
| 3. | 17978-16S-qRTF | ATTAACGCTCGCACCCTCTG | qRT-PCR primer for 16S transcript |
| 4. | BfmS RTP FP | TGTCTGTGTCGAAGACGACG | qRT-PCR primer for *bfmS* transcript |
| 5. | BfmS RTP RP | CAGATGCACGTGTACGGCTA | qRT-PCR primer for *bfmS* transcript |
| 6. | BfmR RTP FP | GCGCGTATTCGTGCTTTGTT | qRT-PCR primer for *bfmR* transcript |
| 7. | BfmR RTP RP | GTTCAACGTTACCGAACGGC | qRT-PCR primer for *bfmR* transcript |
| 8. | RpoE RTP FP | TACGTGCGTGGGAAGGTTTT | qRT-PCR primer for *rpoE* transcript |
| 9. | RpoE RTP RP | AGTCCCTGAATTGCTCGATGA | qRT-PCR primer for *rpoE* transcript |
| 10. | CsuAB_RT_F | CGATGCTGCACGTACAAACC | qRT-PCR primer for *cusAB* transcript |
| 11. | CsuAB_RT_R | GGTGTACCTGTGTTTGGAGC | qRT-PCR primer for *csuAB* transcript |
| 12. | CsuE_RT_F | GCAACCAACGAGCCATGAAG | qRT-PCR primer for *csuE* transcript |
| 13. | CsuE_RT_R | ACTCGACCAACGCTCGTTAC | qRT-PCR primer for *csuE* transcript |
| 14. | CsuD_RT_F | GTTGAACAACGGATAGCGGC | qRT-PCR primer for *csuD* transcript |
| 15. | CsuD_RT_R | GGCATAGGTTTGCCATCTGC | qRT-PCR primer for *csuD* transcript |
| 16. | Omp33_RT_F | AAGTGTTGGCGCTACTTTCG | qRT-PCR primer for *omp33* transcript |
| 17. | Omp33_RT_R | GCGGAATTCACCAAGGTTGT | qRT-PCR primer for *omp33* transcript |
| 18. | CarO_RT_F | TGGCAATCGGTTTTGAAGCTG | qRT-PCR primer for *carO* transcript |
| 19. | CarO_RT_R | CGAAAGTAGCGCCAACACTT | qRT-PCR primer for *carO* transcript |
| 20. | DcaP_RT_F | GCTATGCAGAAGGCAGAGGT | qRT-PCR primer for *dcaP* transcript |
| 21. | DcaP_RT_R | GTCAGTACCAACTGCAACGC | qRT-PCR primer for *dcaP* transcript |
| 22. | OprD_RT_F | CGACTGGTCCAGCTGATGAA | qRT-PCR primer for *oprD* transcript |
| 23. | OprD_RT_R | ACCGCTGTTCTGTTGGTACG | qRT-PCR primer for *oprD* transcript |
| 24. | pET-WT_PscI_F | GGACATGTCTAAAGGTCAAACTTTACAAG | Primers for cloning WT *A. baumannii* Hfq STOP codon into pET-28 between NcoI and XhoI sites. [NcoI and PscI are isocaudomers] |
| 25. | pET-WT_Xho_R | AACTCGAGTTAACGATTGTTTTCGTCGTC | Primers for cloning WT *A. baumannii* Hfq with STOP codon into pET-28 between NcoI and XhoI sites. [NcoI and PscI are isocaudomers] |
| 26. | pET-66_Xho_R | AACTCGAGACGAGCTGGAACAACTGTAGA | Primers for cloning *A. baumannii* Hfq_66_ with STOP codon into pET-28 between NcoI and XhoI sites. [NcoI and PscI are isocaudomers] |
| 27. | pET-NG0_invF | GTTGTTCCAGCTCGTATAACGATTCTAAATTTGAA | Infusion Primers for cloning *A. baumannii* Hfq_NG0_ with STOP codon into pET-28 using pET-WT plasmid as template. |
| 28. | pET-NG0_invR | TTTAGAATCGTTATCACGAGCTGGAACAACTGTAGA | Infusion Primers for cloning *A. baumannii* Hfq_NG0_ with STOP codon into pET-28 using pET-WT plasmid as template. |
| 29. | pET-NG1_invF | CAAGGTGCTGGCTTCGATAACGATTCTAAATTTGAA | Infusion Primers for cloning *A. baumannii* Hfq_NG1_ with STOP codon into pET-28 using pET-WT plasmid as template. |
| 30. | pET-NG1_invR | TTTAGAATCGTTATCGAAGCCAGCACCTTGAGCACCACC | Infusion Primers for cloning *A. baumannii* Hfq_NG1_ with STOP codon into pET-28 using pET-WT plasmid as template. |
| 31. | pET-T0_invF | CATCAAGGCGGTTTTTAACTCGAGCACCACCACCACC | Infusion Primers for cloning *A. baumannii* Hfq_T0_ with STOP codon into pET-28 using pET-WT plasmid as template. |
| 32. | pET-T0_invR | GTGGTGCTCGAGTTAAAAACCGCCTTGATGTCCGCC | Infusion Primers for cloning *A. baumannii* Hfq_T0_ with STOP codon into pET-28 using pET-WT plasmid as template. |
| 33. | pET-AbEc_invF | TTACGCAAGACAAAGGAGATCCGGCTGCTAACAAAG | Infusion Primers for cloning *A. baumannii* Hfq_AbEc_ with STOP codon into pET-28 using pET-WT plasmid as template. |
| 34. | pET-AbEc_invR | CGGTTTCTTCGCTGAAAACCGCCTTGATGTCCGCCGAA | Infusion Primers for cloning *A. baumannii* Hfq_AbEc_ with STOP codon into pET-28 using pET-WT plasmid as template. |
| 35. | EC-Ctip-inf-F | CATCAAGGCGGTTTTGACAGCGAAGAAACCGAATAA | Infusion Primers for introducing the *E. coli* Hfq C-tip with STOP codon into pET-28 using *E. coli* gDNA as a template. |
| 36. | EC-Ctip-inf-R | TAGCAGCCGGATCTCCTTTGTCTTGCGTAAAATAGA | Infusion Primers for introducing the *E. coli* Hfq C-tip with STOP codon into pET-28 using *E. coli* gDNA as a template. |
| 37. | AprF-Bam | ATCAGGATCCGTCGACCTGCAGTTC | Apramycin gene amplification for cloning knock out construct in pUC 18 plasmid |
| 38. | AprR-Kpn | ATGGTACCGTGTAGGCTGGAGCTGCTTC | Apramycin gene amplification for cloning knock out construct in pUC 18 plasmid |
| 39. | FPUS500hfq_Sal1 | ATGTCGACATTGCAGGCAGAACAACC | Cloning primers for *hfq* Upstream 500 bp |
| 40. | RPDS500hfq_BamH1 | ATTGGGATCCACCTTTAGACATTTTTAACTCC | Cloning primers for *hfq* Upstream 500 bp |
| 41. | FPDS500hfq_Kpn1 | GTGGGTACCCGTTAATTGATTAGCTTGAA | Cloning primers for *hfq* Downstream 500 bp |
| 42. | RPDS500hfq_EcoR1 | GCGGCGAATTCCAAACCTATCCC | Cloning primers for *hfq* Downstream 500 bp |
| 43. | Hfq-CDS_KI-F | GGAGTTAAAAGGATCATGTCTAAAGGTCAAACTTTACAAG | Insertion of the *hfq* CDS for all the variants with pET-28 harbouring variants as a template |
| 44. | Hfq-CDS_KI-R | GCAGGTCGACGGATCCCGGATCTCAGTGGTGGT | Insertion of the *hfq* CDS for all the variants with pET-28 harbouring variants as a template |
| 45. | Hfq US 150 FP | CTTACTTGACGATAAAGCAGGC | Primers for PCR construct of Hfq variants to be cloned into the genome |
| 46. | Hfq DS 150 RP | CAGCAAATATTAATACCATTTGCAAAGAA | Primers for PCR construct of Hfq variants to be cloned into the genome |
| 47. | Hfq US 150-b FP | CCGGATCCCTTACTTGACGATAAAGCAGGC | Primers for cloning Hfq locus from clinical strains into pUC18 |
| 48. | Hfq DS 150-b RP | CCGGATCCCAGCAAATATTAATACCATTTGCAAAGAA | Primers for cloning Hfq locus from clinical strains into pUC18 |
| 49. | Hfq CDS FP | TCTAAAGGTCAAACTTTACAAG | Primers to screen for successful Hfq knock-in in *A. baumannii Δhfq* genome |
| 50. | Hfq CDS RP | GACTGGTTTTTTTCAAGCTAATC | Primers to screen for successful Hfq knock-in in *A. baumannii Δhfq* genome |
| 51. | oligoRPT41 | TAGCGGATCCAAGCAAAATTTGCACACTGTC | Forward primer for amplification of *A. baumannii hfq* with its promoter (6). |
| 52. | oligoRPT42 | GCGCGGATCCTTAATATGCTTTTTTATATTTTTAAAGCC | Forward primer for amplification of *A. baumannii hfq* with its terminator (6). |

**Supporting Figures:**


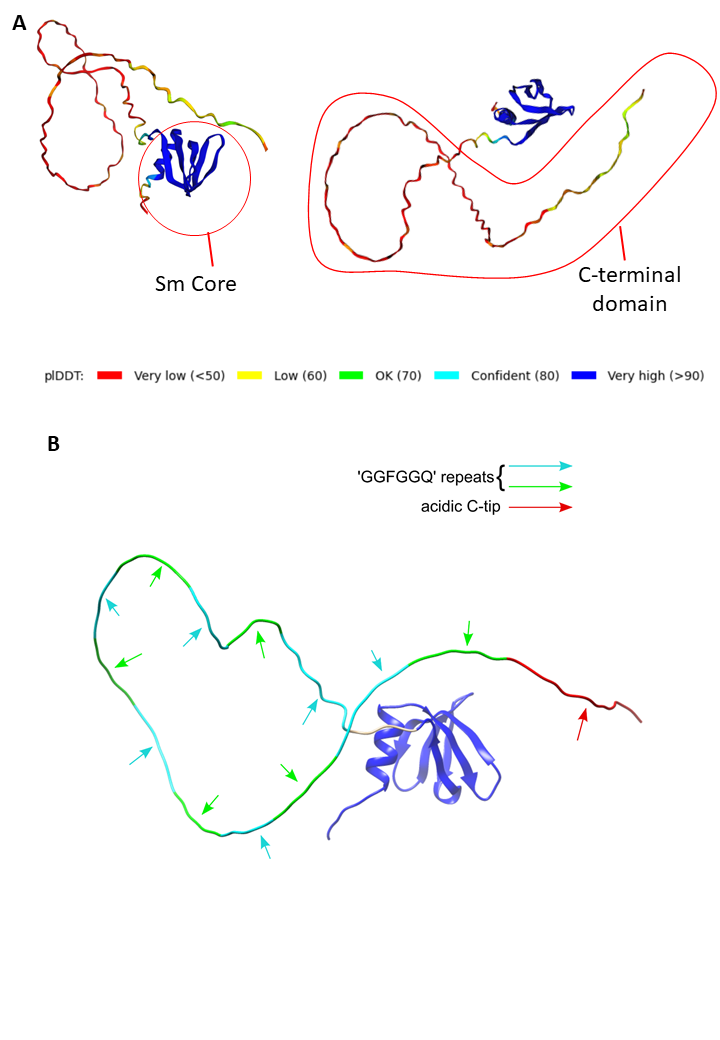


**Figure S1: Structural features of *Acinetobacter baumannii* Hfq protein. A|** *A. baumannii* Hfq monomer predicted by Alphafold2 (ABHfqFL_9b407_0). The RNA binding Sm core and the unusually long intrinsically disordered C-terminal domain (CTD) are highlighted. **B|** The ‘GGFGGQ’ repeats and the acidic C-tip of *A. baumannii* Hfq are highlighted using UCSF Chimera software. Green and Cyan indicates alternate ‘GGFGGQ’ repeats; Red indicates the acidic C-tip.

**
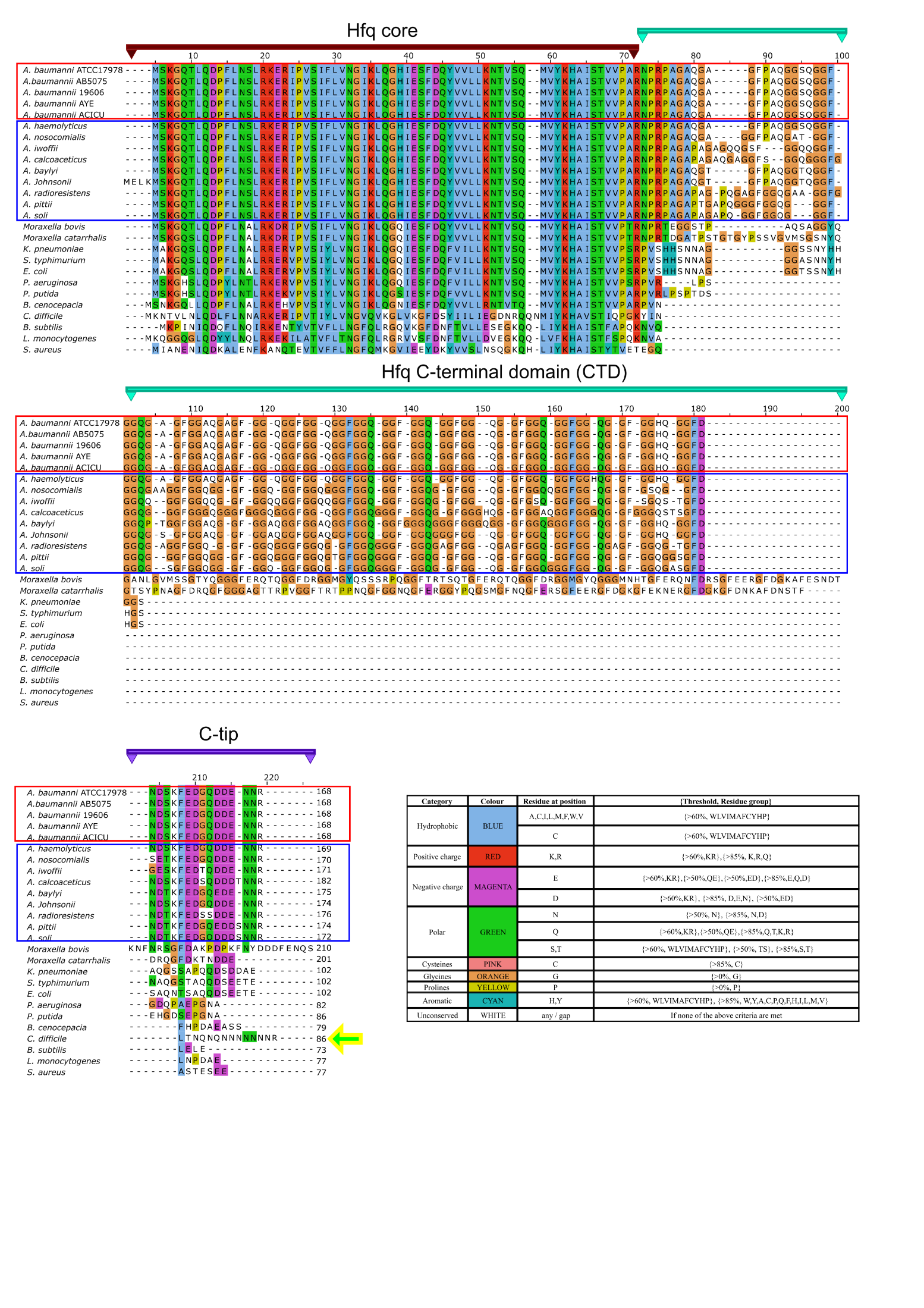
**

**Figure S2: Multiple Sequence Alignment of *A. baumannii* Hfq with Hfq from other Bacteria.** The sequences of Hfq protein were derived from the NCBI database and aligned using EMBL-EBI MUSCLE alignment. The alignment was visualized using Jalview and coloured according to Clustal X default colour scheme (inset). The red box indicates Hfq proteins from different strains of *A. baumannii.* The blue box highlights Hfq proteins from different species of *Acinetobacter.* The arrow points at *C. difficile* Hfq protein that is devoid of any C-terminal acid residues. Different structural features of Hfq are indicated.

**
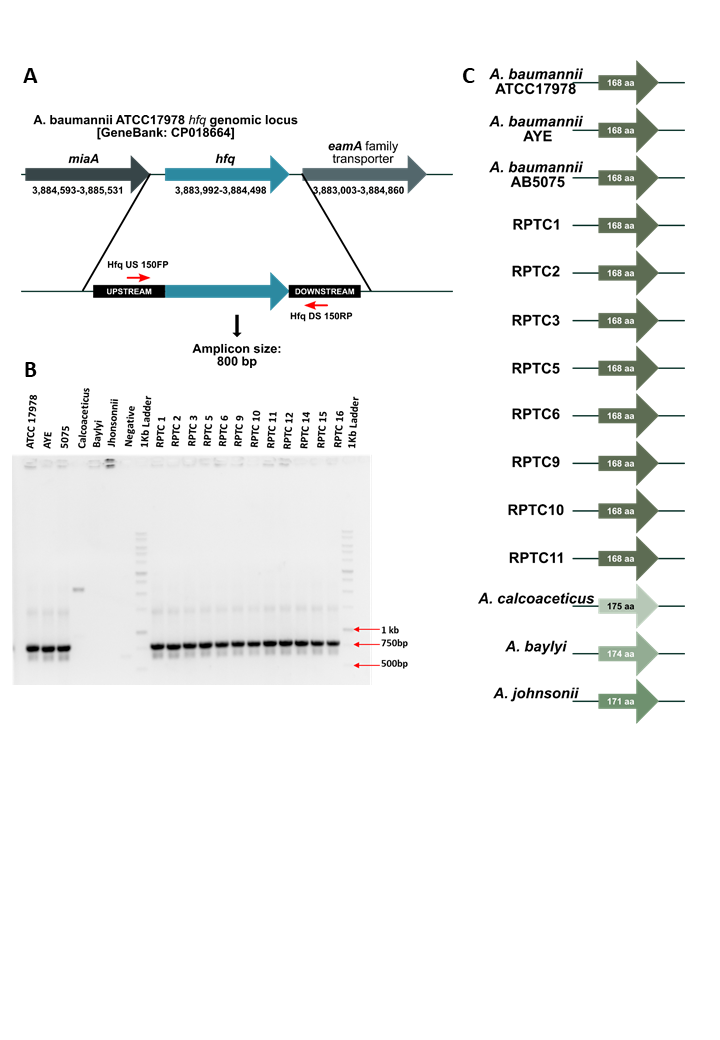
**

**Figure S3: Assessment of Hfq locus conservation among different strains of *A. baumannii*. A**| Genomic location of the *hfq* gene. The location of the *hfq* gene on *A. baumannii* ATCC17978 genome is highlighted in blue and is encompassed by the *miaA* gene on its 5’ upstream region and *eamA* gene on its 3’ downstream region. The amplification product from this region using Hfq US 150 FP and Hfq DS 150 RP PCR primers should result in an amplicon of size of ~800bp. **B**| Screening for presence of Hfq genomic locus. Genomic DNA was isolated from the indicated of *Acinetobacter sp.* strains along with from the *A. baumannii* clinical strains from our strain data bank (RPTC strains) (23). The isolated genomic DNAs were screened for Hfq locus amplicon corresponding to that of *A. baumannii* ATCC17978 strain (WT) using Hfq US 150 FP and Hfq DS 150 RP PCR primers. The PCR products were resolved in a 1% agarose gel and visualized. All the bands that appeared were found to resolve at a region above the 750bp band of the ladder. All the *A. baumannii* strains exhibited the presence of the same Hfq genomic locus. **C**| Assessment of the clinical strains to check for conservation of Hfq coding sequence. The PCR product amplified from eight of the clinical strains using Hfq US 150-b FP and Hfq DS 150-b RP PCR primers were cloned into a pUC18 vector and sequenced. The genomic sequence was translated using an online tool (translate tool -Expasy) and the Hfq protein sequences were compared with the Hfq sequence from NCBI database. All the Hfq proteins from the clinical strains are 100% identical to the *A. baumannii* lab strains (ATCC17978, AYE and AB5075) comprising of 168 amino acids.

**
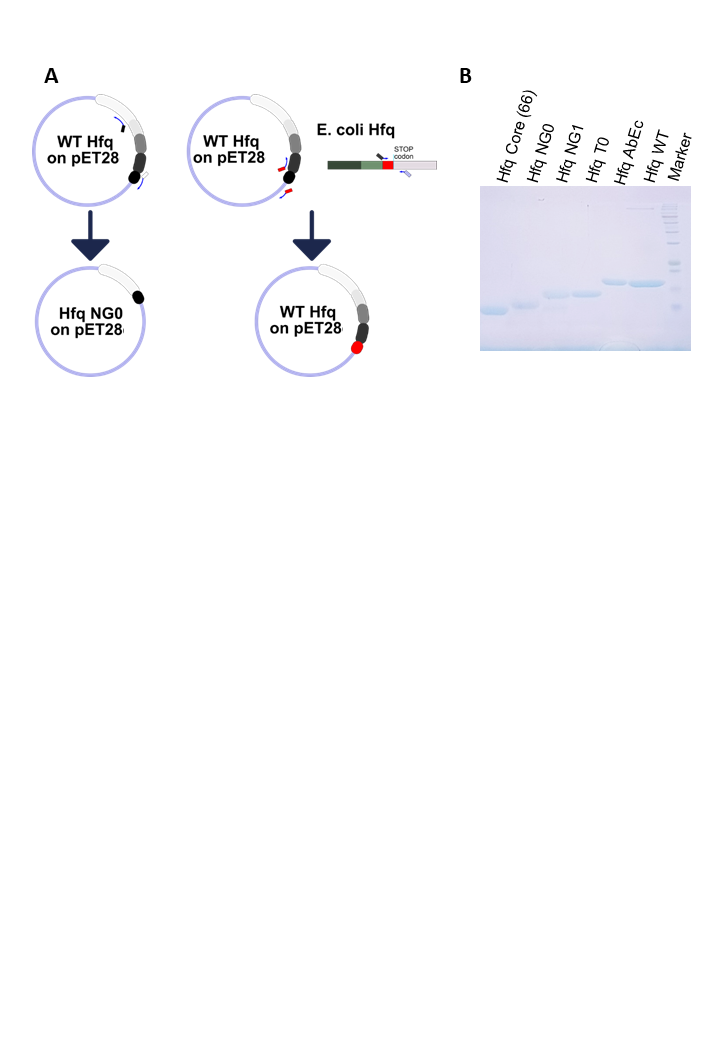
**

**Fig S4: Cloning, expression and purification of *A. baumannii* Hfq variants. A**| Overview of inverse PCR-based cloning of *A. baumannii* Hfq variants. The WT and Hfq_66_ were cloned within NcoI and XhoI sites. All the other Hfq variants (except for AbEc) were constructed with the help of an infusion cloning kit using WT Hfq cloned in pET28c as a template. The plasmid templates were inverse PCR amplified to exclude the genomic region to be removed from the CTD and subsequently ligated. For the AbEc variant, pET-WT and *E. coli* Hfq C-tip were separately linearized using PCR amplification with homologous regions. The PCR products were subsequently ligated using an infusion cloning kit. **B**| The purified Hfq variants resolved on a 15% SDS PAGE Gel.

**
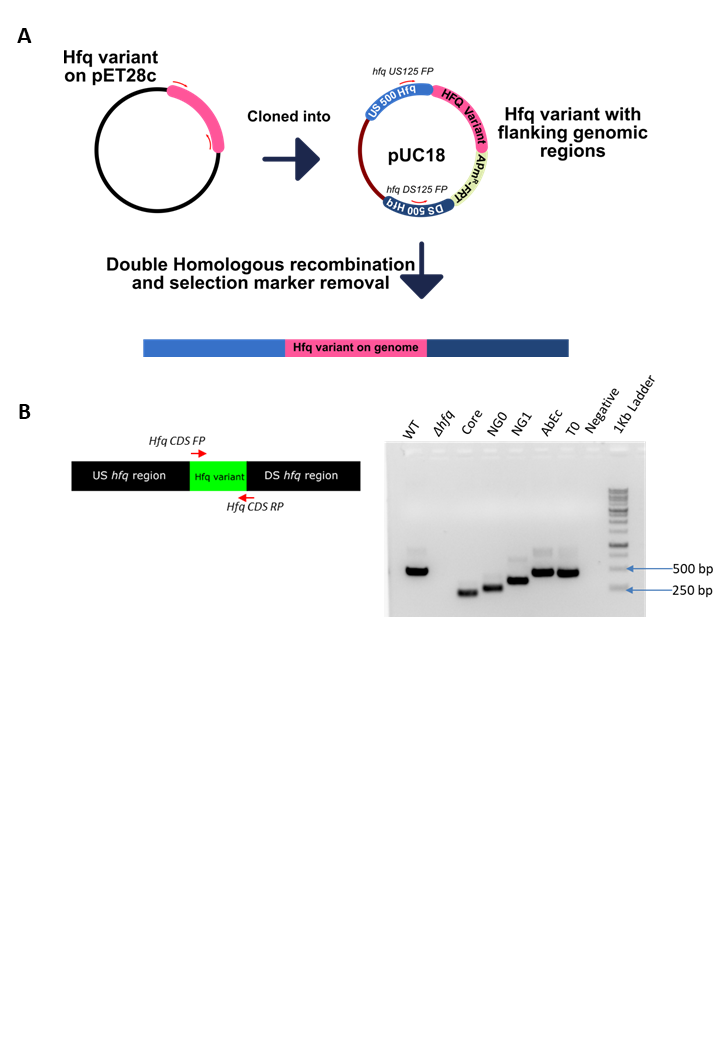
**

**Figure S5: Complementing the Hfq variants into *A. baumannii* cells. A**| Genomic complementation strategy construction of *A. baumannii* Hfq mutants. The Hfq variants on pET28c were amplified and cloned into a PUC18 vector harbouring the upstream (US) 500bp and downstream (DS) 500 bp of Hfq along with an Apramycin selection marker. The construct is then used as a template to generate a PCR product of the Hfq variants with US and DS 125bp homologous regions. This PCR product is then inserted into the Hfq genomic locus of the *A. baumannii Δhfq* strain using a double homologous recombination strategy (PMID: 33593839 and 25096877). **B**| The *A. baumannii* Hfq mutants are confirmed using PCR with primers for the coding region of Hfq. The PCR product is then resolved on a 1% agarose gel.

**
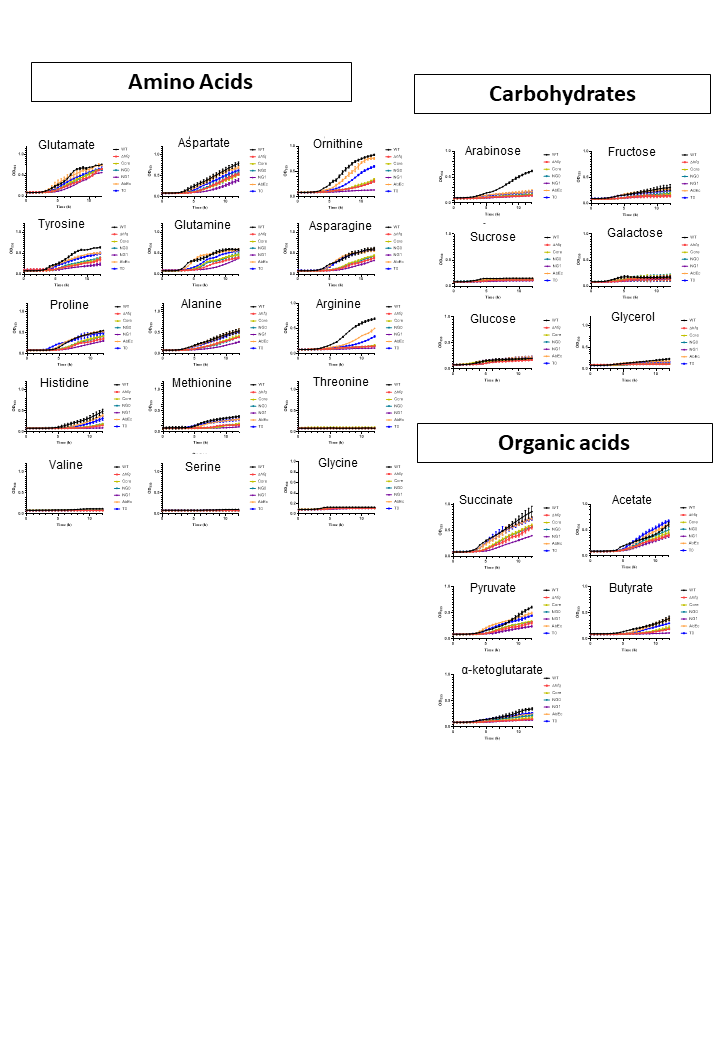
**

**Figure S6: Assessment of growth of the *A. baumannii* strains in single carbon sources.** The *A. baumannii* WT and mutant strains were sub-cultured by diluting 1000-times from 0.4 A_600_ secondary culture into 1X M9 minimal salts supplemented with 10 mM concentration of the indicated carbon source. The cultures were incubated in a 96-well plate at 37 ⁰C with shaking in a microplate reader. The growth was profiled by measuring optical density at 600 nm over the course of 12 hours at 30 min interval (detailed methodology in main text). The data obtained was plotted as A_600_ vs time, where each data point represents four independent values and the error bar represents standard deviation (S.D). Core = Hfq_66._ All the growth phenotype assays are the representative plot of three independent biological replicates.

**
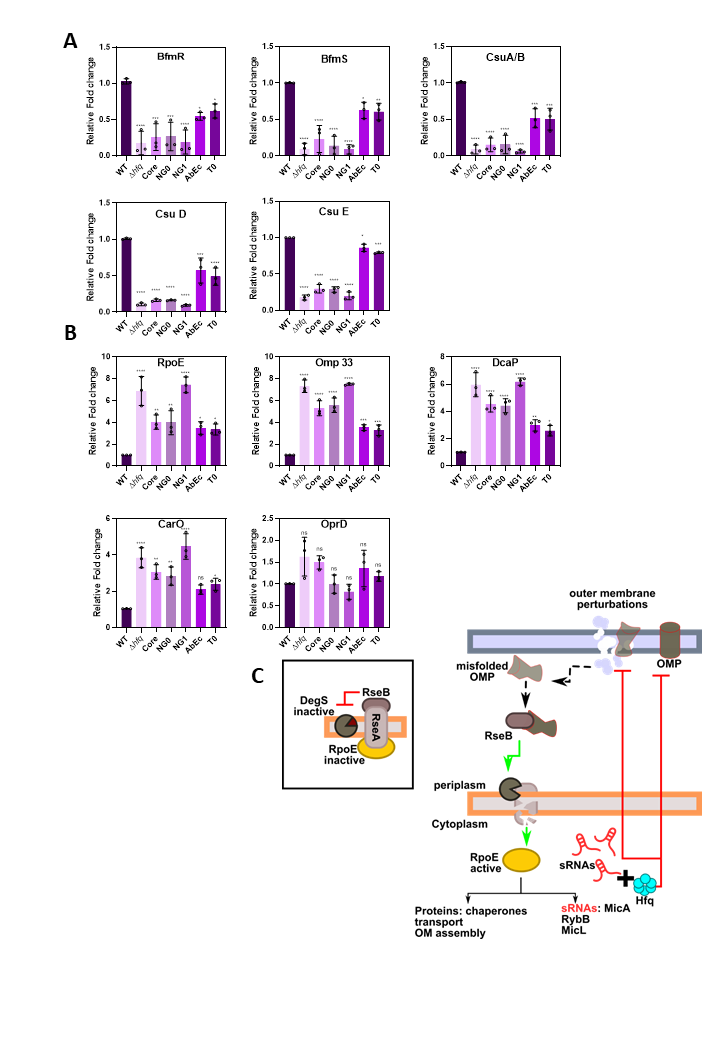
**

**Figure S7: Investigating the effect of Hfq mutations on transcript levels of genes associated with biofilm formation and membrane homeostasis. A**| Quantification of biofilm associated genes. Total RNA was isolated from the *A. baumannii* WT and Hfq mutants to prepare cDNA. The transcript levels of different biofilm associated genes like *bfmR, bfmS, csuA/B, csuD, and csuE* were quantified by qRT-PCR. Each data represents the mean of three independent experiments, and the error bars represent S.D. Statistical significance was determined by one-way ANOVA (p-value was *, p ≤ 0.05; **, p ≤ 0.001; ***, p ≤ 0.001; ****, p ≤ 0.0001; ns, non-significant). Tukey’s test was used as a post hoc test to determine the statistical significance of all pairs of data. **B**| Quantification of transcript levels of membrane homeostasis related genes. The transcript levels of different membrane homeostasis maintaining genes like *rpoE, omp33,* *dcaP, carO,* and *oprD* were quantified by qRT-PCR. Each data represents the mean of three independent experiments, and the error bars represent S.D. Statistical significance was determined by one-way ANOVA (p-value was *, p ≤ 0.05; **, p ≤ 0.001; ***, p ≤ 0.001; ****, p ≤ 0.0001; ns, non-significant). Tukey’s test was used as a post hoc test to determine the statistical significance of all pairs of data. **C**| A schematic of membrane homeostasis pathway in *E. coli.* The loss of outer membrane integrity results in misfolding of different outer membrane proteins (OMPs). These misfolded OMPs binds on to RseB protein activating it. The activated RseB in turn activates DegS proteasome which degrades the membrane bound RseA. The RseA protein keeps the σ^E^ (RpoE) inactive by remaining bound to it, hence, its degradation renders the σ^E^ active. The active σ^E^ induces the transcription of multiple maintenance proteins (coding arm) and different small RNAs or sRNAs (non-coding arm). The upregulated sRNAs like MicA and RybB bind to Hfq to repress OMP levels. This regulation helps in bringing about gradual cessation of the σ^E^-mediated membrane repair pathway so as to prevent constitutive activity of the σ^E^ transcription factor. Core = Hfq_66._

**
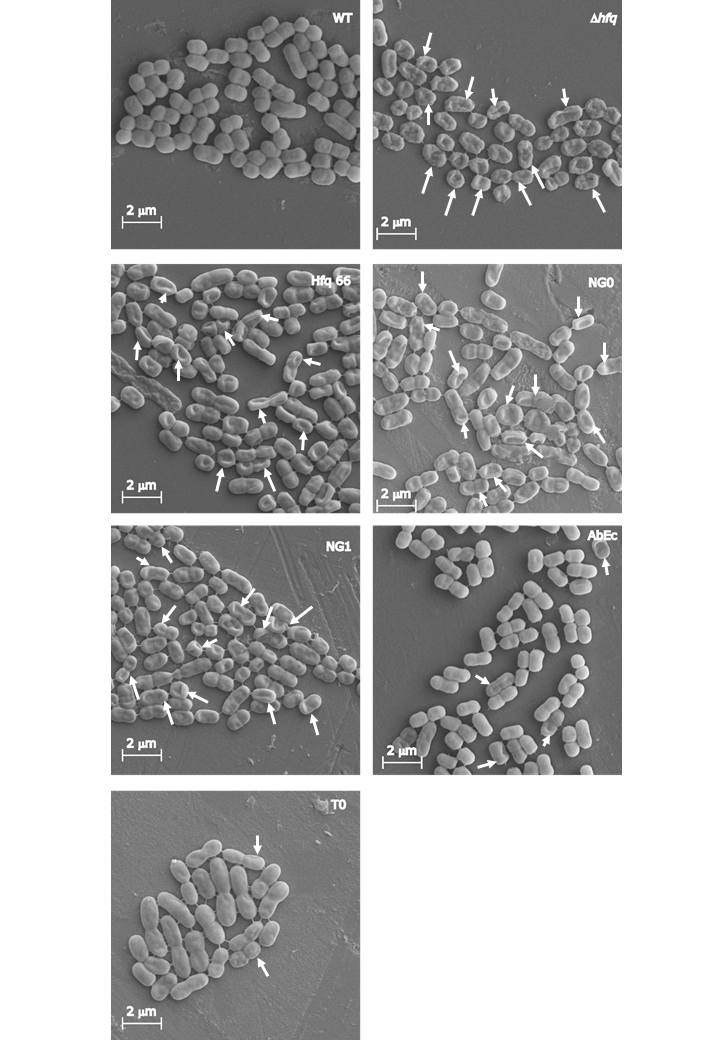
**

**Figure S8: Investigating morphological variations of the *A. baumannii* strains.** Actively growing cells of the different strains of *A. baumannii* were sub-cultured and grown till mid-log phase (A_600_= 0.6). The cells were then washed twice and resuspended in sterile 1X PBS and fixed using a glutaraldehyde-formaldehyde mix. The cells were subsequently dehydrated using an increasing concentration of ethanol and visualized post-gold coating at 10000X magnification using a scanning electron microscope. The scale bar corresponds to a length of 2µm, and the white arrows indicate pit formation and misshapen *A. baumannii* cells.

**
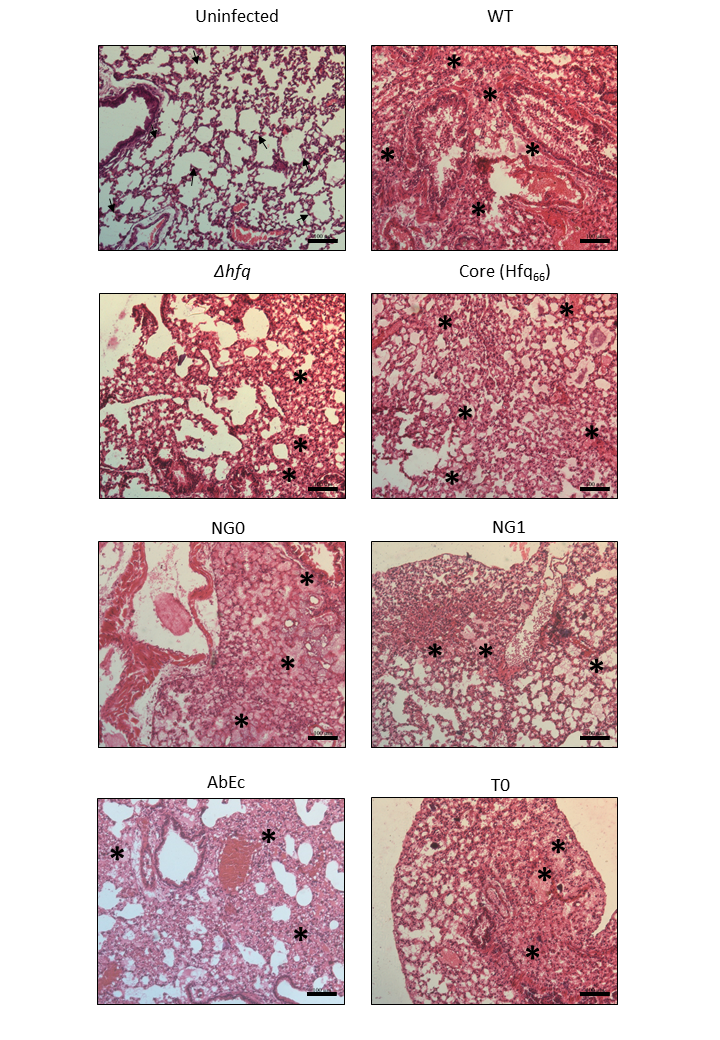
**

**Figure S9: Evaluating the of *A. baumannii* strains to cause pneumonia in mice.** BALB/c mice were infected intranasally with the different strains of *A. baumannii.* Lungs isolated from infected and uninfected mice was visualized by performing tissue histology. Lung histology was performed using haematoxylin and eosin staining in the mice pneumonia model after 36 hours of infection. Black asterisk (*****) denotes regions of alveolar pus aggregation along with shrinkage in alveolar space, and black arrow (**↑**) indicates alveolar spaces in a healthy lung tissue from an uninfected mouse. The black scale bars indicate 100 µm length.

**Supporting References:**

1. Sharma, A., Dubey, V., Sharma, R., Devnath, K., Gupta, V. K., Akhter, J., Bhando, T., Verma, A., Ambatipudi, K., Sarkar, M., and Pathania, R. (2018) The unusual glycine-rich C terminus of the Acinetobacter baumannii RNA chaperone Hfq plays an important role in bacterial physiology. *J. Biol. Chem.* **293**, 13377–13388

2. Basu, P., Elgrably-Weiss, M., Hassouna, F., Kumar, M., Wiener, R., and Altuvia, S. (2021) RNA binding of Hfq monomers promotes RelA-mediated hexamerization in a limiting Hfq environment. *Nat. Commun.* 10.1038/s41467-021-22553-x

3. Baba, T., Ara, T., Hasegawa, M., Takai, Y., Okumura, Y., Baba, M., Datsenko, K. A., Tomita, M., Wanner, B. L., and Mori, H. (2006) Construction of Escherichia coli K-12 in-frame, single-gene knockout mutants: The Keio collection. *Mol. Syst. Biol.* 10.1038/msb4100050

4. Dubey, V., Gupta, R., and Pathania, R. (2021) Targeting superoxide dismutase confers enhanced reactive oxygen species-mediated eradication of polymyxin b-induced acinetobacter baumannii persisters. *Antimicrob. Agents Chemother.* 10.1128/AAC.02180-20

5. Sharma, A., Sharma, R., Bhattacharyya, T., Bhando, T., and Pathania, R. (2017) Fosfomycin resistance in Acinetobacter baumannii is mediated by efflux through a major facilitator superfamily (MFS) transporter-AbaF. *J. Antimicrob. Chemother.* **72**, 68–74

6. Tucker, A. T., Nowicki, E. M., Boll, J. M., Knauf, G. A., Burdis, N. C., Stephen Trent, M., and Davies, B. W. (2014) Defining gene-phenotype relationships in acinetobacter baumannii through one-step chromosomal gene inactivation. *MBio*. **5**, 1–9

7. Večerek, B., Rajkowitsch, L., Sonnleitner, E., Schroeder, R., and Bläsi, U. (2008) The C-terminal domain of Escherichia coli Hfq is required for regulation. *Nucleic Acids Res.* **36**, 133–143

8. Santiago-Frangos, A., Jeliazkov, J. R., Gray, J. J., and Woodson, S. A. (2017) Acidic C-terminal domains autoregulate the RNA chaperone Hfq. 10.7554/eLife.27049.001
